# Partially Recursively Induced Structured Moderation (PRISM) for modeling racial differences in endometrial cancer survival

**DOI:** 10.1371/journal.pone.0268221

**Published:** 2023-01-31

**Authors:** J. Sunil Rao, Erin Kobetz, Huilin Yu, Jordan Baeker-Bispo, Zinzi Bailey

**Affiliations:** 1 Department of Public Health Sciences, University of Miami, Miami, Florida, United States of America; 2 Department of Medicine, University of Miami, Miami, Florida, United States of America; 3 Sylvester Comprehensive Cancer Center, University of Miami, Miami, Florida, United States of America; PLOS (Public Library of Science), UNITED KINGDOM

## Abstract

**Purpose:**

Health disparities are driven by a complex interplay of determinants operating across multiple levels of influence. However, while recognized conceptually, much disparities research fails to capture this inherent complexity in study focus and/or design; little of such work accounts for the interplay across the multiple levels of influence from structural (contextual) to biological or clinical. We developed a novel modeling framework that addresses these challenges and provides new insights.

**Methods:**

We used data from the Florida Cancer Data System on endometrial cancer patients and geocoded-derived social determinants of health to demonstrate the applicability of a new modeling paradigm we term PRISM regression. PRISM is a new highly interpretable tree-based modeling framework that allows for automatic discovery of potentially non-linear hierarchical interactions between health determinants at multiple levels and differences in survival outcomes between groups of interest, including through a new specific area-level disparity estimate (SPADE) incorporating these multilevel influences.

**Results:**

PRISM demonstrates that hierarchical influences on racial disparity in endometrial cancer survival appear to be statistically relevant and that these better predict survival differences than only using individual level determinants. The interpretability of the models allows more careful inspection of the nature of these hierarchical effects on disparity. Additionally, SPADE estimates show distinct geographical patterns across census tracts in Florida.

**Conclusion:**

PRISM can provide a powerful new modeling framework with which to better understand racial disparities in cancer survival.

## Introduction

Cancer disparities are created and maintained through a complex interplay of factors on multiple levels of influence. Numerous models have conceptually organized the multiple levels of health determinants, both in the context of illness broadly [1–3] and along cancer continuum specifically [4–6]. For example, Lynch and Rebbeck’s Multi-level Biological and Social Integrative Construct integrates macroenvironmental- (e.g. health policy, neighborhood exposures, and family structure), individual- (e.g. behavior and sociodemographic) and biological-levels (e.g. genome, tissue and cell features) to assess cancer etiology and outcomes [6]. Understanding factors on each of these levels as well as the interaction between them is essential for comprehensively characterizing the etiology of cancer health disparities across time and place. Although conceptual models acknowledging hierarchical social determinants of cancer outcomes are widely accepted, existing statistical models are limited in their capacity to capture the multi-level nature of such determinants and their complex interplay with more proximal prognostic factors [4, 6].

For instance, although overall incidence of endometrial cancer (EC) is highest among White women, Black women suffer higher mortality and worse survival from EC than do their White counterparts [7–9]. Black women have more high-RISK tumors (which encompasses both type and grade) and more late stage disease, regardless of type [10]. Although diagnosis with Type II EC is likely partially a function of genetic predisposition [11], racial survival disparities persist after controlling for clinical disease characteristics like grade and histology [12]. Poor EC survival outcomes in Black women thus reflect more than the racial patterning of prognostic factors associated with disease biology; they likely also reflect the social-environmental context in which disease occurs, and how that context interacts with individual-level disease features. Existing studies that attempt to account for the contribution of social determinants to EC survival disparities typically do so by controlling for individual indicators, like patient socioeconomic status (SES) or health insurance or aggressive histology, in traditional Cox proportional hazards models, without integrating the interaction between multiple levels of factors likely driving these disparities [8, 13, 14]. In contrast, tree-based statistical methods can capture hierarchies between health determinants as well as interactions across levels, without relying on linear methods, which may not reflect the true relationships between them. However, to our knowledge, these interactions have not been articulated methodologically.

In this paper, we present a novel statistical approach that aims to accomplish this within the context of cancer health disparities, specifically those related to EC survival. Our tree-based approach, termed PRISM, will help identify population sub-groups at greatest risk of contributing to excess disease burden by modeling the hierarchical effects of neighborhood-level determinants of EC survival and their complex interactions with individual-level disease features. In turn, this will provide a more precise characterization of disparities and strategies for reduction.

## Methods

### Ethics statement

All data are fully anonymized in the Florida Cancer Data System (FCDS) and thus were fully anonymized before we accessed them.

This study utilizes data from the Florida Cancer Data System (FCDS), a statewide incident cancer registry that provides ongoing surveillance of new cancer cases from diagnosis to death. In total, 320 hospitals report nearly 115,000 new cases annually. The FCDS is designed to collect systematic data on the clinical attributes of disease. Our cohort consisted of female Non-Hispanic White (NHW) and Non-Hispanic Black (NHB) endometrial cancer cases in FCDS from 2005 to 2014. Our primary outcome of interest was overall survival measured in days from date of diagnosis. We included the following individual-level characteristics in these analyses based on data in the FCDS: (a)race; (b)marital status; (c)insurance type; (d)age at diagnosis; (e)histologic type; (f)grade; (g)stage; and (h)course of treatment.

Race was operationalized as an indicator variable corresponding to identifying as non-Hispanic Black (NHB). We categorized the patient’s marital status at the time of primary diagnosis into one of five categories: married, separated, single, unmarried or domestic partner, and widowed. Insurance type consists of the patient’s primary method of payment or insurance coverage at the time of initial diagnosis and/or treatment, categorized as Private, Medicaid, Medicare, Military/VA/Tricare/HIS, Uninsured, or Insured, type not specified. Age at diagnosis was considered as continuous variable measured in years. Histologic type identifies the microscopic anatomy of cells, is a basis for staging and the determination of treatment options, and affects the prognosis and course of the disease. We included four categories for these analyses: carcinosarcoma, endometrioid adenocarcinoma, uterine serous carcinoma, and undifferentiated endometrial cancer. The grade of the tumor describes the resemblance of the tumor to normal tissue. Cancers were coded as grade 1 through 4, where higher grades reflect less cell differentiation. Cancers were classified as stage1–4 at diagnosis as defined in FCDS by the American Joint Committee on Cancer Staging Manual, 6th edition [15]. Furthermore, rather than using a hybridized variable defined by combining stage and grade (to address redundancy), we used stricter histology and grade criteria for inclusion. For example, we excluded non-endometrioid histologies with either missing or misclassified/inconsistent grade codes (e.g. coded 1 or 2), and defined endometrioid using only three histologies [16]. Three binary indicators for radiation, chemotherapy, and surgery reflect whether the treatment was delivered as part of a patient’s first-course therapy. Receipt of surgery was defined as receipt of hysterectomy (partial or complete).

In recent years, geocodes for patients’ census tract at diagnosis have been included which enables characterization of neighborhoods by key social determinants. In these analyses, we considered the following census-tract-level variables that were extracted from American Community Survey (ACS) based on their potential for mediating, moderating, driving, and/or confounding racial disparities in cancer: (a) median household income, (b) GINI coefficient (a measure of income inequality), (c) percent of individuals living below the poverty line, (d) percent of individuals 16+ in civilian labor force who are unemployed, (e) percent of adults 25+ with less than a high school education, (f) percent of housing units with more than 1 resident per room, (g) percent of housing units with no access to a vehicle, and (h) percent of housing units that are renter-occupied. Our goal was to understand racial differences in cancer-free survival and the moderating effects that might play a role in these differences. For simple descriptive analyses to compare the predictors by race, we used linear regression for continuous variables, multinomial regression for categorical variables, and ordinal regression for ordinal variables. We then developed a new modeling framework described below for understanding the multilevel moderation of the effect of race by individual level and contextual level variables discussed above.

## Model development

In order to fit hierarchical interactions between individual level variables and contextual ones, we first introduce a generalized surface varying coefficient model. Let *T* be the logarithm of the failure time and **x** = (*x*_1_, …, *x*_*p*_)′ be a *p*-dimensional covariate vector of *focus* variables and **z** a *K*-dimensional vector of individual level variables. When *T* is subject to right censoring, we observe (*y*, *δ*, **x**, **z**) with *y* = *min*(*T*, *C*), where *C* is the logarithm of the censoring time and *δ* = 1{*T* ≤ *C*} is the censoring indicator. We assume that a random sample {*y*_*im*_, *δ*_*im*_, **x**_*i,m*_, **z**_*im*_}; *i* = 1, …, *n*_*m*_, *m* = 1, …, *M* where *M* represents the total number of tracts and *n*_*m*_ the number of observations in tract *m* from the parent distribution has been collected. We will assume the relationship between *y* and **x** and **z** follows the model,

$$\begin{array}{c} y=x^{\prime }\beta \left(z\right)+e, \end{array}$$ (1)

where *β*(**z**)′ = (*β*_1_(**z**), …, *β*_*p*_(**z**)). The *e* are errors with an unknown distribution. Here the individual level variables **z** are seen to be moderating the effect of **x** on *y*. The true underlying form for *β*_*j*_(**z**); *j* = 1, …, *p* is an unknown complex *p*-dimensional hypersurface which modulates the effect of each *x*_*j*_. We call this a *surface-varying coefficient model*. This is a multivariate analog of the varying coefficient models introduced by [17].

### PRISM approximation and recursive partitioning

Estimating the model above directly proves challenging in higher dimensions and thus our structured approximation takes the form for each *β*_*j*_(**z**) as,

$$\begin{array}{c} \beta_{j}\left(z\right)=\sum_{l=1}^{L}\theta_{jl}I\left(z\in A_{l}\right). \end{array}$$ (2)

Where *A*_*l*_ is a partition of the predictor space determined by **z**. All *A*_*l*_ are assumed mutually exclusive. This model which we term the *Partially Recursively Induced Structured Moderation (PRISM)* regression approximation to (1) defined as,

$$\begin{array}{c} y=\sum_{j=1}^{p}x_{j}\sum_{l=1}^{L}\theta_{jl}I\left(z\in A_{l}\right)+e, \end{array}$$ (3)

where *θ*_*jl*_ are the partition (node)-specific regression parameter estimates. So this approximation fits separate linear models with focus variables **x** to observations in each *A*_*l*_ whose membership in which are determined by individual level variables **z**. This is done using a weighted least squares approach to account for censoring. For each *A*_*l*_, let ${\hat{F}}_{nl}$ be the Kaplan-Meier estimator of the distribution function *F* of *T* for observations in *A*_*l*_. Following [18] and Stute and Wang (1993), we can write ${\hat{F}}_{nl}=\sum_{i\in A_{l}}d_{ni}\left(y_{\left(i\right)}\leq y\right)$ where the *d*_*ni*_ are the Kaplan-Meier weights representing the jump points in the Kaplan-Meier estimator. Specifically, *d*_*n*1_ = *δ*_(1)_/*n* and $d_{ni}=\delta_{\left(i\right)}/\left(n-i+1\right)\prod_{j=1}^{i-1}{\left(\left(n-j\right)/\left(n-j+1\right)\right)}^{\delta_{\left(j\right)}};i\in A_{l}$.

Contextual level variables **w** can be further incorporated into the structured moderation model as *β*_*j*_(**z**(**w**)). This allows for multilevel moderation of the effect of **x** on *y* by now both **z** and **w** and we term this model hierarchical PRISM or HPRISM. We use a tree-structured regression approach to fit the model [19]. As is well known, there are many advantages of this approach over traditional multilevel models based on linear (mixed) models. These tree-based models generally good predictors for complex data. They are non-linear, non-parametric, resistant to outliers and missing values and because of their piecewise structure, provide ease of interpretation. In addition, they naturally find complex interactions which may not be known apriori. When considering multilevel moderation of the effect of **x**, these can also be used as exploratory tools from which post-specified linear mixed models can be fit.

In order to generate an empirical fit HPRISM models, using our sample {*y*_*i,m*_, *δ*_*im*_, **x**_*im*_, **z**_*im*_, **w**_*m*_}; *i* = 1, …, *n*_*m*_, *m* = 1, …, *M* where *M*, we use a recursive partitioning approach as follows: For ease of discussion, take for example *p* = 1, *x* = *race*, *z*_1_, …, *z*_*K*_ and a single contextual level variable *w* = *percentpov* (i.e. a census tract level social determinant) and $n=\sum_{m=1}^{M}n_{m}m$ observations. Consider *z*_*k*_ and all observed values *z*_*k*1_, …, *z*_*kn*_. Index the root node as *τ* that pools all of the observations. That is, (*x*_*i*_, *y*_*i*_, *w*_*i*_), $i=1,\ldots ,\sum_{m=1}^{M}n_{m}m$, and with some abuse of notation, $w^{\prime }=\left\{{\left(w_{1}\right)}_{n_{1}},\ldots ,{\left(w_{M}\right)}_{n_{M}}\right\}$. Consider a split *z*_*ki*_ < *s* that generates daughter nodes *τ*_*L*_ and *τ*_*R*_. Then define the change in residual sum of squares (RSS) for this split as,

$$\begin{array}{c} \Delta RSS\left(s\right)=\sum_{i\in \tau }{\hat{r_{i}}}^{2}-\left\{\sum_{i\in \tau_{L}}{\hat{r_{i}}}^{2}+\sum_{i\in \tau_{R}}{\hat{r_{i}}}^{2}\right\}. \end{array}$$

where $\hat{r_{i}}=\Delta_{i}r_{i}+\left(1-\Delta_{i}\right)E\left(e|e>r_{i}\right)$ with Δ_*i*_ being the censoring indicator and *r*_*i*_ = *y*_*i*_ − *θ*_0_ − *θ*_1_ *x*_*i*_ − *θ*_2_ *x*_i_*w_*i*_, *E*(*e*|*e* > *r*_*i*_) is estimated as the mean value of all residuals of uncensored observations greater than *r*_*i*_ [20].

Then choose the split value among all *z*_*k*_’s that maximizes the Δ*RSS*(*s*). *Recurse* till a stopping rule is satisfied (see below). This can be visualized as a *binary tree* consisting of a set of terminal nodes whose corresponding branches are the recursively applied splitting rule from the root node onwards.

#### Other potential splitting rules

Log-rank test statistic is a widely used splitting criteria. To accommodate log-rank splitting in our scenario, we can calculate the log-rank test statistic for both daughter nodes with respect to the focus variable (use focus variable to group observations in each daughter node). Then we take the absolute value of the difference between the log-rank test for two daughter nodes and use this value as the splitting criteria. The best split is determined by searching for the splitting variable *z*_*k*_ and *s* that maximize the absolute difference. Besides, other statistics such as Uno’s C [21] and Harrell’s C [22] can be used to form splitting criteria as well.

#### Exclusion criteria

Since all censored observations will get zero weights, only the uncensored observations need to be considered. For each potential split, there should be uncensored observations in both right and left daughter nodes representing the different racial groups, and within each racial group, the uncensored observations should not come from the same census tract. Thus, this will allow estimation of both the main effect of race and interaction between race and the tract level social determinant post-splitting.

### Stopping rule and estimating tree size

A variety of stopping rules or tree size estimation strategies can be borrowed from the CART literature. For instance, one can stop splitting when the number of observations in each daughter node being larger than some minimal number. One can also impose a restriction that splitting only be allowed when the overall tree goodness of fit criterion improves by some minimal margin. The *C*_*p*_ method (reference) is such a rule where typical values of the complexity parameter *C*_*p*_ are set at 0.01 thus indicating that any split that does not decrease the overall lack of fit by this factor will not be attempted. The optimal choice of *C*_*p*_ is open to scrutiny but techniques like cross-validation could be used to estimate this. Finally, tree pruning where a generously grown tree is pruned backwards via weakest link cost complexity pruning can be implemented. This also requires something like cross-validation and can be computationally prohibitive. In our case, given the more complex nature of the tree-growing procedure, we chose to set a user-defined value of *C*_*p*_ at 0.01 (as is the default say in the rpart R package).

### Evaluating predictive performance

All PRISM and HPRISM models were evaluated for their predictive performance. The FCDS cohort was stratified by censoring status and then split by strata into an 80% training set and a 20% test set. Models were built on the training set and then test observations were fed down each tree based on their *x* and *w* values, and a predicted value of the log survival time was estimated from the terminal nodes that each test observation fell into. This process was repeated 100 times and the mean empirical test set Harrell’s C statistic [22] standard error (SE) were reported. Harrell’s C statistic is a widely accepted measure of predictive performance based on validation data that may be subject to right censoring. C statistics are routinely used in the medical literature to quantify the capacity of an estimated risk score in discriminating among subjects with different event times. It provides a global assessment of a fitted survival model rather than focusing on the prediction for a fixed time.

### Local variable importance (Lvimp) and interpreting the trees

As will be shown, the tree models can be quite complex and challenging to interpret directly. This is because it’s natural to focus on the overall tree topologies rather than the terminal nodes themselves which represent the fitted models. Here we will develop customized strategies for better interpretation. Variable importance was originally designed for tree-based models using measures involving surrogate variables [19]. Other measures based on mean overall improvement in node impurity for a tree have also been proposed. One interesting such measure is called variable importance (vimp) [23] which uses a prediction error approach involving “noising-up” a variable at a time and examining the difference in prediction error when a variable is noised-up by permuting its value randomly, compared to prediction error under the original predictor. Variables with large vimp values are ranked highly in terms of variable importance. In our setting however, we want to understand the individual level variables that are driving each terminal node—so a *local* vimp (Lvimp). In order to do this, we must condition on the observations in a terminal node while we apply the noising-up procedure variable by variable and re-building the entire tree. Differences in (weighted) prediction errors are evaluated only using those observations in the terminal node of interest.

Beyond the obvious usefulness of the Lvimp measures in terms of understanding the variables most important in determining a particular subgroup (i.e. terminal node), the Lvimp values can also be used to better visualize the relative “distance” between terminal nodes in a tree. This can be done using an old graphical tool for visualizing multivariate data called the Andrews curve [24]. Generically, Andrews curve start with a vector *c* which is a high dimensional datapoint where *c* = {*c*_1_, *c*_2_, …, *c*_*d*_} in *R*^*d*^. We can then define the finite Fourier series:

$$\begin{array}{c} f_{c}\left(t\right)=\frac{c_{1}}{\sqrt{2}}+c_{2}sin\left(t\right)+c_{3}cos\left(t\right)+c_{4}sin\left(t\right)+c_{5}cos\left(t\right)+\ldots . \end{array}$$

By substituting in the rank-orderd Lvimp measures for each terminal node for *c*, we can generate an Andrews curve for each splitting path (branch) which defines a terminal node.

### Specific area-level disparity estimation (SPADE)

Understanding how disparity is distributed and (hierarchically) moderated within a geographical area at the tract level is of great interest. Direct estimation at the tract level is not possible because all individuals in a tract share the same value of the social determinant. However, the HPRISM model allows such an estimate to be reverse engineered. Notice that within each terminal node, we have estimated $\theta_{l}^{\prime }=\left(\theta_{0l},\theta_{1l},\theta_{2l}\right)$ locally and each terminal node consists of a mixture of observations from different census tracts (areas). Thus, for *x* = 0 if White and *x* = 1 if Black, the predicted mean log survival difference (i.e. disparity) for observations in a terminal node is ${\hat{d}}_{l}=\left(\hat{y}|x=1\right)-\left(\hat{y}|x=0\right)=\left({\hat{\theta }}_{0l}+{\hat{\theta }}_{1l}+{\hat{\theta }}_{2l}*w_{l}\right)-{\hat{\theta }}_{0l}$.

To go back to the tract level itself, gather all of the observations from tract *m* from each terminal node and form the weighted average ${\hat{d}}_{m}=\sum_{l}\eta_{lm}d_{lm}$. The weights *η*_*lm*_ can be made flexible but typically correspond to the relative proportions of individuals from a given tract in a terminal node. We call this the *specific area-level disparity estimate or SPADE*. We can then plot these as a *heat map* by census tract over the state of Florida. In order to combine all individual SPADEs together, we generated a *composite* SPADE. This will give an indication of the effect of considering all social determinants together. The simplest composite estimate simply is to sum the individual SPADEs. This can be shown to yield a somewhat crude but still useful upper bound to the true composite quantity.

## Results

There were 13,506 cases in this derived cohort of which over 11% (1558) were NHB. There are significant differences between racial groups with respect to age, Medicaid and not insured status, frequency of undergoing a hysterectomy, frequency of receiving chemotherapy, marital status in a number of categories, frequency of histology revealing endometrioid adenocarcinoma, stage and grade of tumor.

Table 1 shows overall differences between BNH and WNH for each individual level variable. All p-values were derived from a chi-squared test of homogeneity between races across levels of the variable except for Marital Status and Histology which required use of Fisher’s Exact test due to small expected cell numbers. For Age which is a continuous variable in the modeling, we have displayed quartile groups in Table 1. All p-values are very small indicating strong racial differences. Tables 2 and 3 drill down further and explore racial differences for each level of categorical individual variables. Once again, the majority of p-values are highly significant. From Table 3, for Grade, for WNH versus BNH, the log odds of observing grade 1 versus grade 2 or higher grades is increased by 0.713. For Stage, for WNH versus BNH, the log odds of observing Stage 1 versus Stage 2 or higher is increased by 0.478. For Table 2, the coefficients are interpreted as: for WNH versus BNH, the log odds of being in that category versus in the reference category is increased or decreased by the estimated coefficient where the reference groups of Insurance, Surgery, Chemo, Marital Status and Histology are insured, NOS, Hysterectomy, Chemo, Divorced and Carcinosarcoma respectively. S1 Table of the S1 File gives the list of social determinants of health that were extracted from American Community Survey (ACS) data by linking geocoded census tracts from FCDS.

**Table 1:** Overall racial differences across individual level variables.

Age	21–57	58–64	65–72	73–99	p-value		
WNH	3198	2912	2949	2889	6.254e-11		
BNH	455	437	414	252			
Grade	1	2	3	4	p-value		
WNH	5260	3915	2362	411	<2.2e-16		
BNH	491	410	556	101			
Stage	1	2	3	4	p-value		
WNH	8588	1119	1675	566	<2.2e-16		
BNH	968	164	276	150			
Surgery	Hysterectomy	No hysterectomy	p-value				
WNH	11582	366	4.108e-09				
BNH	348	1210					
Chemo	Chemo	No chemo	p-value				
WNH	1744	10204	2.687e-15				
BNH	348	1210					
Insurance	Insured, NOS	Medicaid	Medicare	Military/VA/Tricare/IHS	Not insured	Private	p-value
WNH	561	305	5621	229	436	4796	< 2.2e-16
BNH	48	120	640	33	109	608	
Marital Status	Divorced	Married	Separated	Singel	Unmarried or Domestic Partner	Widowed	p-value
WNH	1307	6679	73	1907	8	1974	4.998e-4
BNH	205	574	31	463	0	285	
Histology	Carcinosarcoma	Endometrioid Adenocarcinoma	Serous	Undifferentiated	p-value		
WNH	219	11103	613	13	4.998e-4		
BNH	77	1280	199	2			

**Table 2:** Racial differences by variable level.

Variable	Race	SE	p-value
Insurance			
Medicaid	1.525722	0.185008	<2e-16
Medicare	0.285725	0.156063	0.06713
Military/VA/Tricare/IHS	0.521337	0.239341	0.02939
Not Insured	1.072292	0.184614	6.311e-09
Private	0.393163	0.156424	0.01196
Surgery			
No hysterectomy	0.69756	0.11939	5.135e-09
Chemo			
No chemo	-0.520425	0.066117	3.509e-15
Marital Status			
Married	-0.601614	0.086805	4.188e-12
Separated	0.996007	0.227156	1.162e-05
Single	0.436920	0.091254	1.685e-06
Unmarried or Domestic Partner	-9.389655	97.646494	0.9234
Widowed	-0.082848	0.098278	0.3992
Histology			
Endometrioid Adenocarcinoma	-1.115089	0.13573	<2e-16
Serous	-0.079794	0.155595	0.6081
Undifferentiated	-0.826477	0.771006	0.2837

**Table 3:** Racial differences by grade and stage levels.

Grade	Estimate	SE	p-value
Race	0.713177	0.050234	<2e-16
1|2	-0.226224	0.018192	<2e-16
2|3	1.167751	0.020975	<2e-16
3|4	3.344260	0.045970	<2e-16
Stage			
Race	0.478276	0.054722	<2e-16
1|2	0.941912	0.020296	<2e-16
2|3	1.462799	0.022933	<2e-16
3|4	2.948071	0.039310	<2e-16

**Fig 1:**
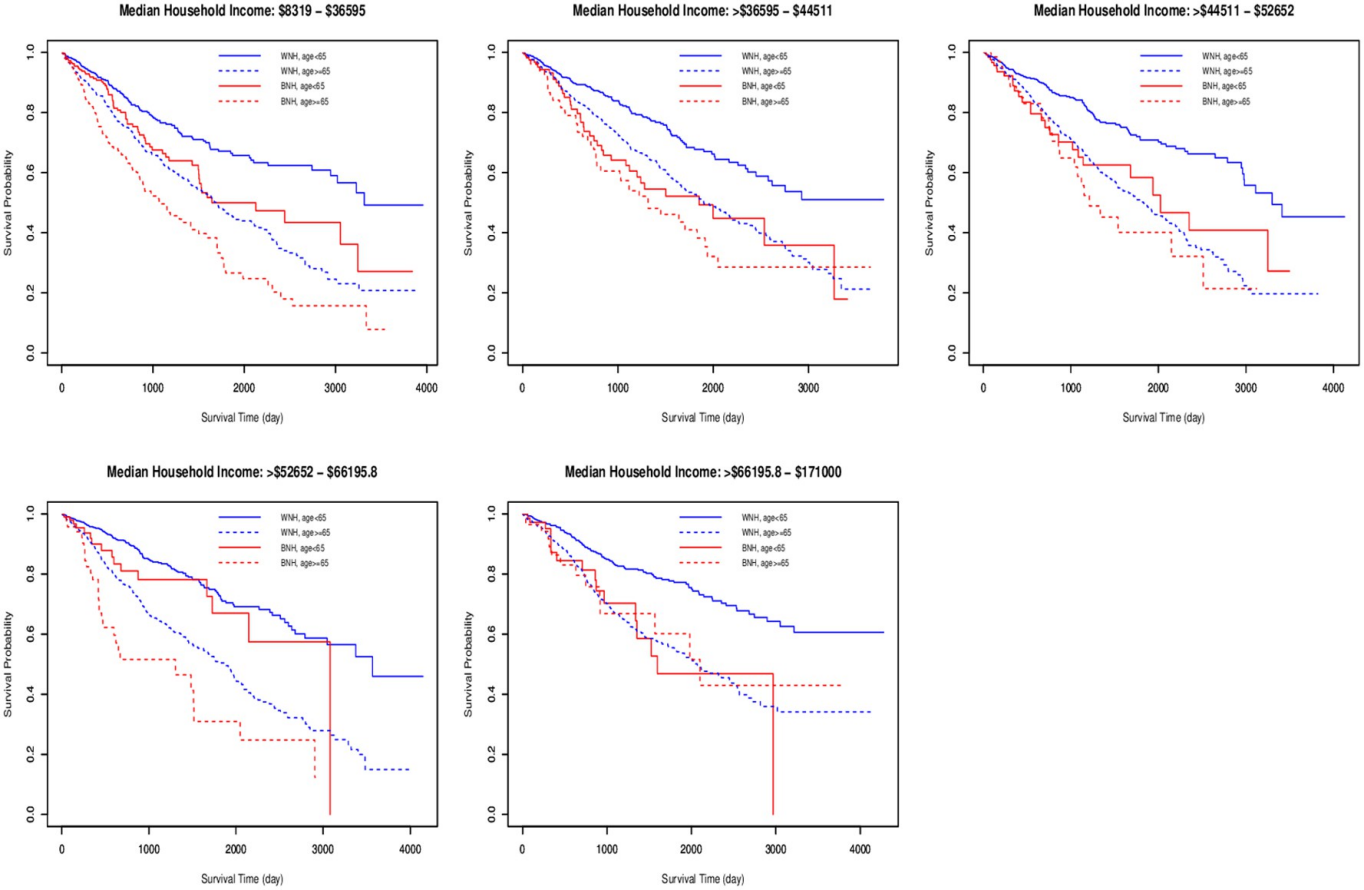
Racial differences in survival broken out by age and MedIncome. Plotted are Kaplan-Meier curves for WNH (blue) and BNH (red).

Kaplan-Meier survival curves were plotted in S1 Fig (S1 File) for our analytic sample comparing NHB and NHW. Of the 13,506 cases in our cohort, 11019 were censored (81.5%) for overall survival. As expected, non-Hispanic Black women with endometrial cancer face poorer survival than non-Hispanic White women, reflected in a widening gap between the corresponding curves (*p* < 0.0001). Fig 1 illustrates the multilevel nature of interactions between race and individual level and area level variables. Kaplan-Meier curves broken out by age-specific racial groups are paneled by ranges of census tract level median income levels. Since the difference between these curves across panels is not constant, this reflects a multilevel interaction. Similar discoveries are made with other social determinants (not shown).

**Fig 2:**
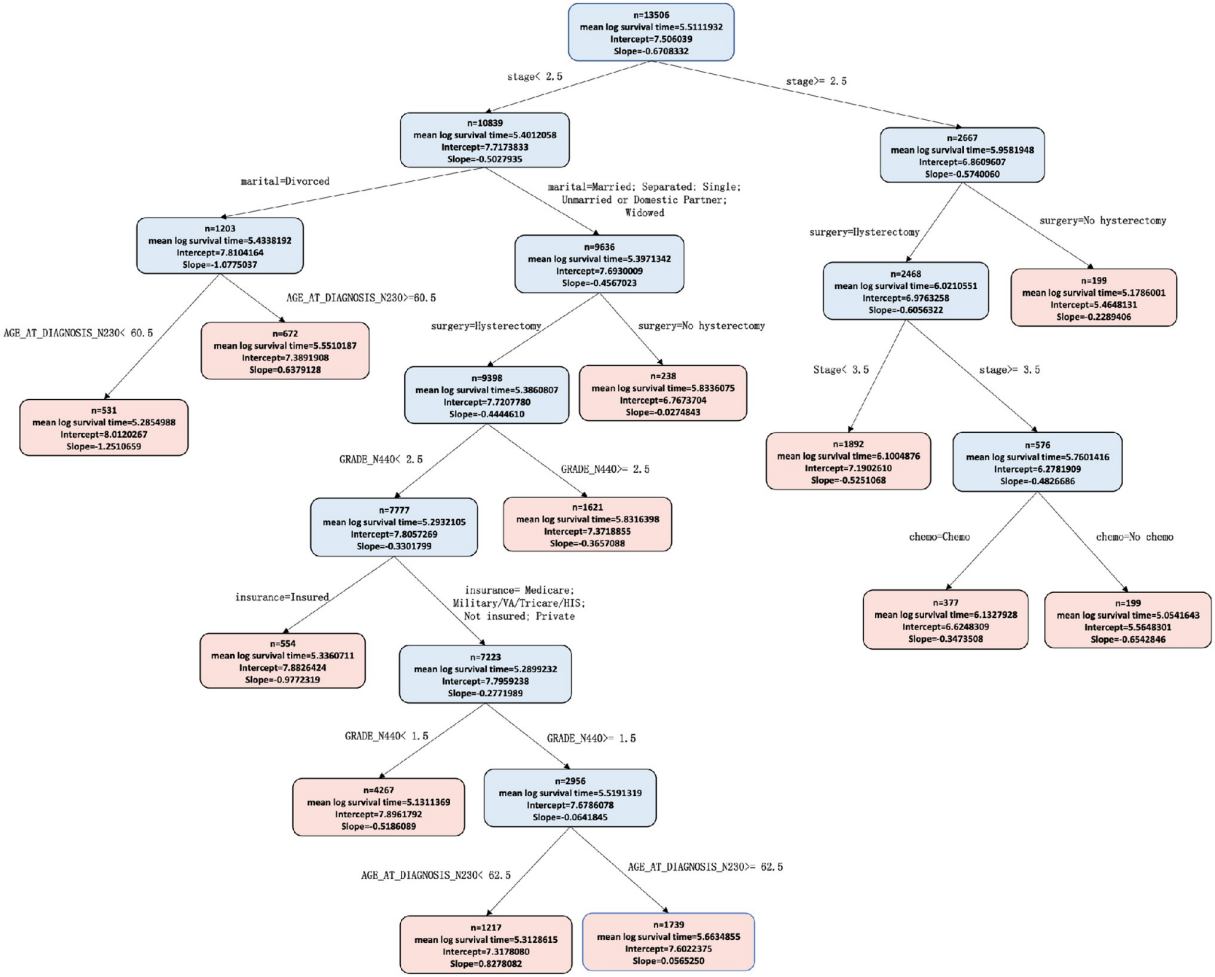
PRISM tree. Inside each terminal node of the PRISM tree is printed sample size, mean log survival time, the intercept and slope effect for race.

**Fig 3:**
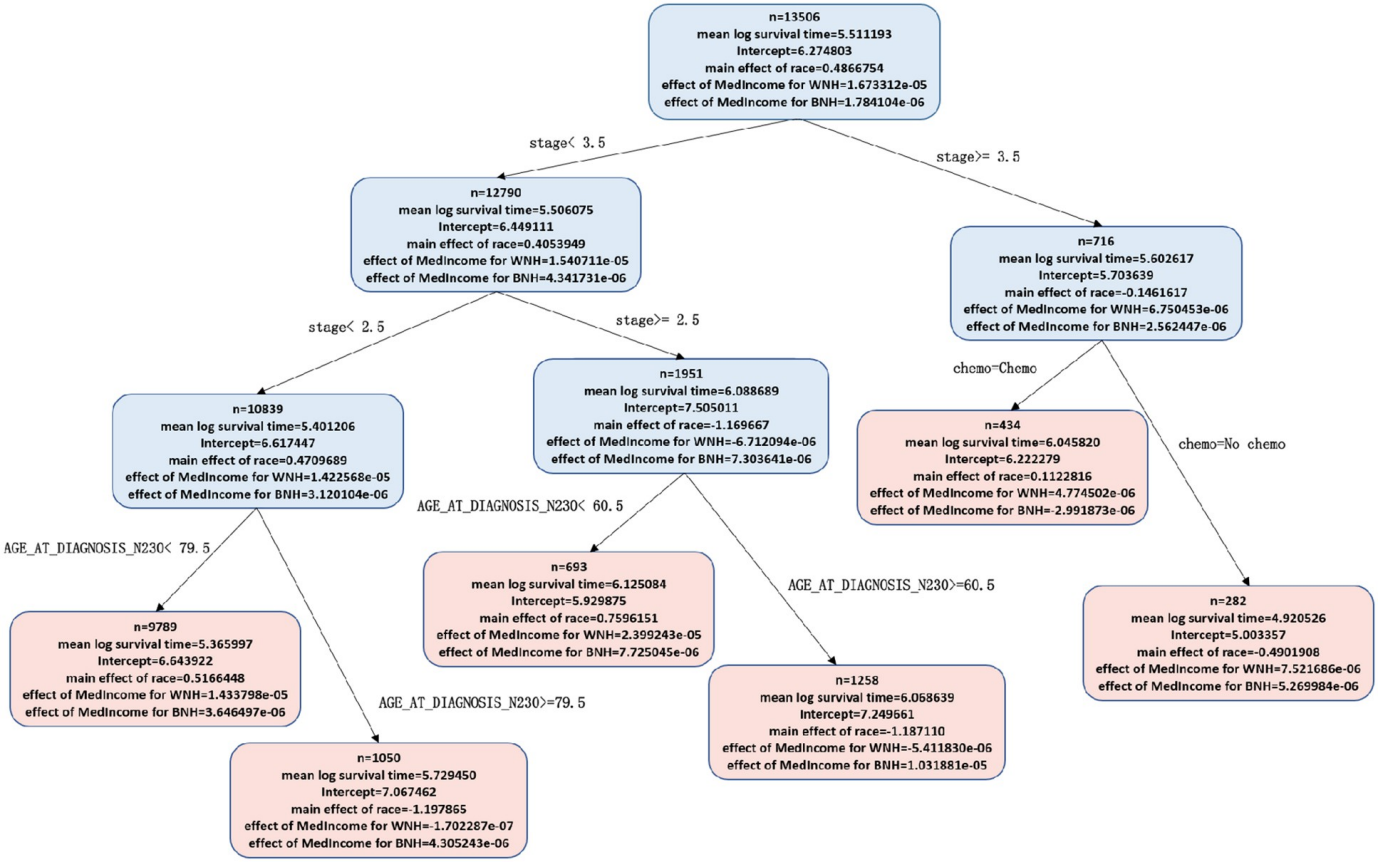
HPRISM tree for MedIncome. Inside each node, the HPRISM tree additionally adds the MedIncome slopes by racial group to each node.

Figs 2 and 3 show the PRISM and HPRISM median income fits respectively (as depicted by tree-like topological graphs) to our cohort. Each node is split at a value of one of the individual level variables and observations are sent to the left or right resulting daughter nodes (colored blue) according to whether they affirmatively obey the split rule or not and this process is recursively repeated until the stopping rule is invoked, at which point an observation reaches a terminal node (colored pink). Within each node is printed the mean log survival time, the intercept, race effect and additionally the interaction of race with tract median income (for the HPRISM tree). Each terminal node represents a discovered subgroup where the racial effect on survival is moderated significantly differently from the root node. These do not represent the same simple subgroups shown in Fig 1; Fig 1 is only a descriptive plot that says that hierarchical moderation may be occurring but does not discover the complex underlying nature that might fully be at play.

When examining the trees more carefully, it is quite clear that Stage and Age at Diagnosis are potentially important variables for the HPRISM tree and marital status, type of insurance coverage and surgery additionally show up for the PRISM tree. We will later show how Lvimp measures will help with interpreting the tree branch paths and formally allow us to rank order the variable importance of each of the individual level variables for each subgroup (terminal node). Other HPRISM trees are in the S1 File (S2a–S2d Fig).

One thing that is noticeable is that some of the HPRISM trees appear topologically simpler than the PRISM tree. It turns out that there is a combined mathematical and operational reason for this when it happens. A sketch goes as follows: call the HPRISM tree *T*_*H*_ and PRISM tree *T*_*P*_. Then, we know that in the root node *τ*_*root*_,

$$\begin{array}{c} RSS{\left(T_{H}\right)}^{\tau_{root}}<RSS{\left(T_{P}\right)}^{\tau_{root}}, \end{array}$$

because HPRISM model includes extra interaction term with *w*. This is the case at each step of splitting. In addition, the total number of split points for a given splitting variable is also smaller for HPRISM due to the split point exclusion criteria. These two facts taken together, imply that the number of splits possible before stopping can likely be less for HPRISM than PRISM. It is customary to expect p-values for the node-specific interaction estimates as a measure of their significance. However, it is well known that these p-values are overly optimistic (small) because they do not account for the amount of fitting done in building the tree. Correcting for such optimism is not easy and relies on approximate distribution theory based on estimates of model complexity. Another option would be to try and use a bootstrap-based approach as was done for the case of phylogenetic tree analysis [25, 26].

Adequacy of model using estimated test set predictive fit was done via Harrell’s c statistic as described in the Methods section. Table 4 shows Harrell C comparisons of the PRISM model versus the various HPRISM models along with accompanying standard error estimates. Increases in empirical test set Harrell C statistic were seen for all HPRISM models over the PRISM model. This provides unbiased validation of the fact that multilevel determinants at the individual and contextual level are indeed important in explaining racial disparity in endometrial cancer survival in the FCDS cohort. When specifically comparing GINI and MedIncome for instance, GINI provides a slightly more predictive information than MedIncome does with Harrell C values of 0.0.531 and 0.576 respectively. Zhu and colleagues [27] used nomograms for predicting cancer-specific and overall survival among patients with EC. They specifically found a predicted C index of 0.782 (95% CI (0.772,0.792)) for overall survival derived from a Cox analysis. Miller and colleagues [28] studied EC recurrence prediction and when using a University of Iowa cohort found very predictive models using both clinical data and also when incorporating genomic predictors (AUC >0.90). However, when they took their models to TCGA data, the AUCs dropped significantly (AUC ≈ 0.60–0.66). Madison and colleagues [13] using SEER data, found that being black, that increased age, aggressive histology and poor tumor grade and advanced stage of disease was associated with increased risk of death. Tejerizo-Garcia and colleagues [29] looked at prognostic factors of overall survival and disease-free survival in a cohort of 276 older patients in Spain. They found that FIGO stage and tumor grade were independent prognostic factors of overall survival in EC patients.

**Table 4:** Test set prediction performance of the various models.

Model	Harrell C Statistic (SE)
PRISM	0.538 (0.012)
HPRISM with GINI	0.531 (0.018)
HPRISM with MedIncome	0.576 (0.021)
HPRISM with PercentCrowd	0.533 (0.016)
HPRISM with PercentNoVeh	0.544 (0.021)
HPRISM with PercentRent	0.552 (0.016)
HPRISM with PercentUnEmp	0.544 (0.019)

**Fig 4:**
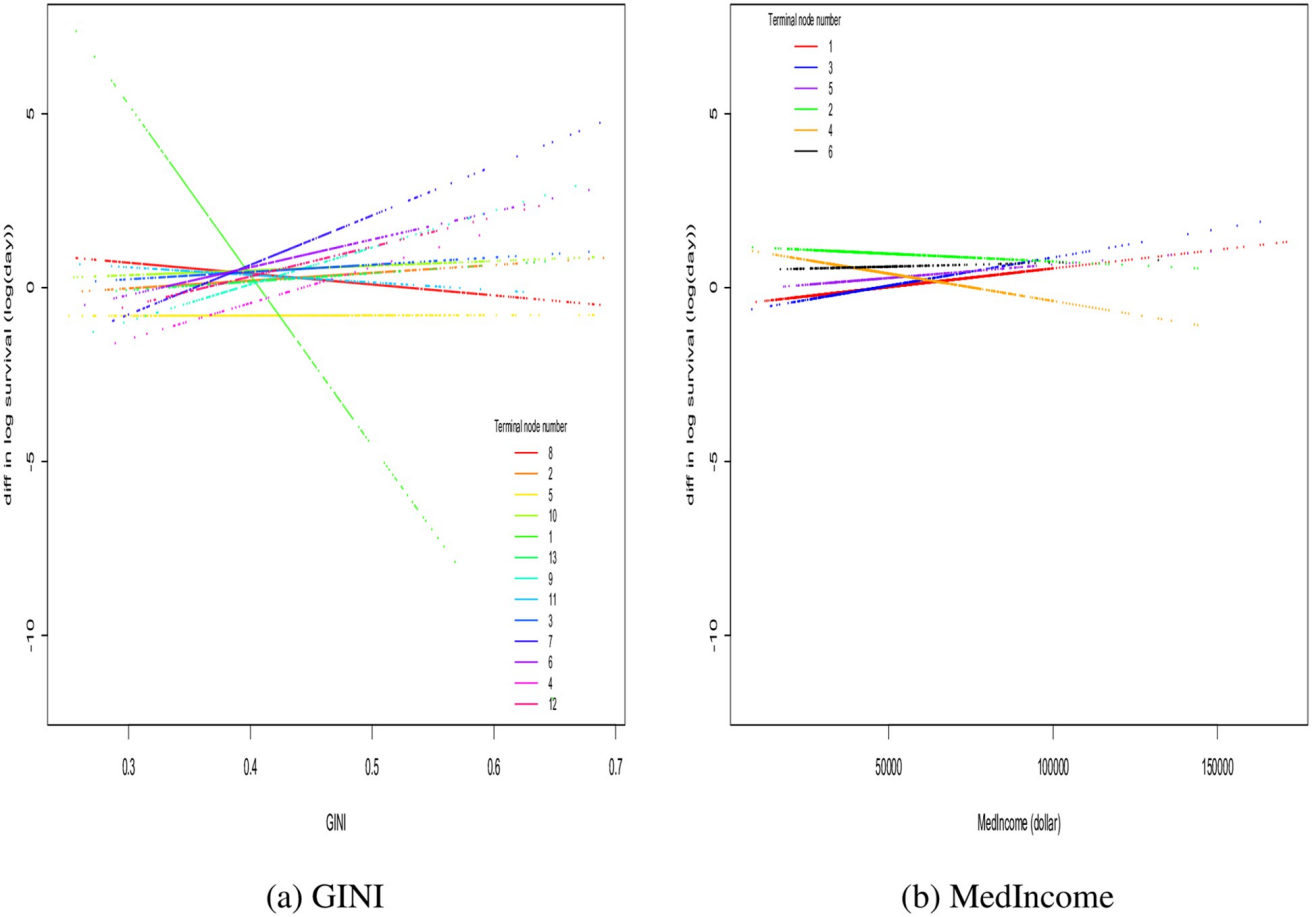
HPRISM hierarchical moderation plots for GINI and MedIncome. Each colored represents a different terminal node (defined by the individual level variables) in the HPRISM tree. The y-axis plots mean difference in log survival between Whites and Blacks at given values of the social determinant. Hierarchical moderation is visible as follows: at the level of individual variables because the y-axis values do not overlap at fixed x-values; at the level of the social determinant, when the lines are not parallel. Notice though for MedIncome, the lines are nearly parallel. (a) GINI. (b) MedIncome.

More can be learned by a deeper examination of the HPRISM tree topologies. Each branch path to a terminal node for an HPRISM tree explicitly details a discovered hierarchical interaction. Fig 4 shows another way to display such interactions. For illustration, plotted are the differences in log survival time between WNH and BNH versus GINI and MedIncome respectively. Each dotted line represents a different terminal node from the HPRISM tree. Conditioning on a particular value of the social determinant variable, we see that the data points on the line do not coincide. This indicates the first layer of interaction (i.e. the interaction of individual level variables defining the terminal nodes with race). The fact the lines are not parallel across different values of GINI is indicative of the hierarchical interaction of race with the individual level variables that define the terminal nodes with the social determinant. Notice though that for MedIncome, the lines are nearly parallel indicating a much weaker hierarchical moderation effect. It then is reasonable to ask why the HPRISM MedIncome tree is topologically different than the PRISM tree. The differences can in part be explained by the different main effect estimates for race high up in the trees which can produce different choices of splitting variables. This initial difference is propagated down the trees. Hierarchical interaction plots for all other HPRISM trees are available in the S1 File.

**Fig 5:**
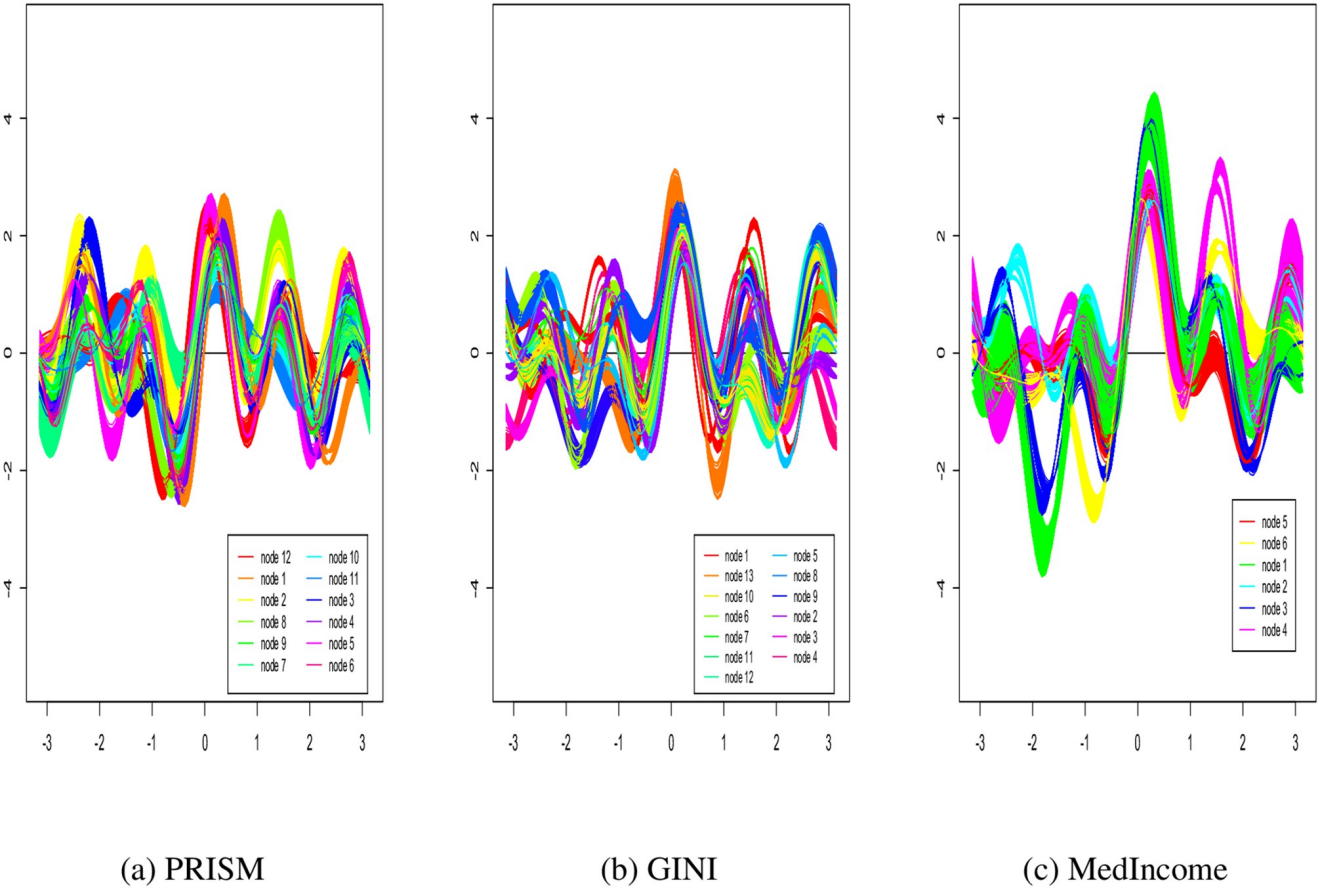
Andrews curves for PRISM and HPRISM models for GINI and MedIncome respectively. (a) PRISM. (b) GINI. (c) MedIncom.

Tables 5 and 6 reports Lvimp ranks for the PRISM tree for each of the MedIncome, GINI HPRISM trees respectively. The rows of the Table give the name of the individual level variable and the columns index terminal node numbers. Table entries show the Lvimp values and their ranks in parentheses. Age appears to be the overwhelmingly most important variable describing BNH-WNH disparity in most terminal nodes for all HPRISM trees, followed by Stage for the MedIncome tree and Stage, Grade and Marital status, Surgery and Insurance for the GINI tree. Note the Lvimp values less than or equal to zero indicate variables that are not important in that branch. Lvimp tables for all other HPRISM trees are available in the S1 File. These Lvimp tables illustrate the moderation effect on an individual-level variable as it’s clear how the rankings across terminal for each variable can change with and without contextual moderation. Fig 5 plots Andrews curves for the PRISM and HPRISM GINI and MedIncome trees to visualize how different or similar the terminal nodes are with respect to racial disparity. Each line represents a unique observation and all observations of the same color are within the same terminal node. Notice how the PRISM tree is less able to find distinct subgroups as either of the HPRISM trees. In particular, the MedIncome HPRISM tree found wider differences across terminal nodes (i.e. different disparity subgroups) than the GINI tree did. Andrews plots for all other HPRISM trees are available in the S1 File (S3 Fig).

**Table 5:** Local variable importance measure for the PRISM tree. T1, …, T12 indicate terminal node number.

Tree.Variable	T1	T2	T3	T4	T5	T6	T7	T8	T9	T10	T11	T12
Prism.Insurance	0.0811(5)	0.4992(6)	-0.2638(4)	-0.0522(4)	0.0000(2)	-0.2622(5)	0.0000(3)	0.0062(6)	0.0000(2)	0.1512(5)	0.0120(4)	0.0000(3)
Prism.Surgery	0.1977(4)	0.9580(4)	-1.0113(9)	0.0000(1)	-0.0588(9)	-0.6953(9)	0.6772(1)	2.2953(1)	-0.0988(8)	0.9961(2)	0.0000(5)	-0.1023(9)
Prism.Radiation	0.0000(9)	0.0000(9)	0.0000(1)	0.0000(1)	0.0000(2)	0.0000(1)	0.0000(3)	0.0000(8)	0.0000(2)	0.0000(8)	0.0000(5)	0.0000(3)
Prism.Chemo	0.0566(6)	0.1702(7)	-0.1312(3)	-0.0810(5)	0.0000(2)	-0.0898(3)	0.0000(3)	0.0703(5)	-0.1608(9)	0.0291(7)	-0.0067(7)	1.0814(2)
Prism.Marriage	0.3209(1)	1.3249(1)	-0.3278(5)	-0.6787(7)	0.0000(2)	-0.1943(4)	0.0000(3)	0.2114(3)	0.0000(2)	0.1555(4)	0.6440(2)	0.0000(3)
Prism.Age	0.0092(8)	0.8429(5)	-0.5734(6)	-1.1744(8)	0.0000(2)	-0.4748(7)	0.0000(3)	0.5939(2)	0.0000(2)	0.3222(3)	0.7314(1)	0.0000(3)
Prism.Grade	0.2345(2)	1.0790(2)	-0.9832(8)	-0.1409(6)	0.0000(2)	-0.5782(8)	0.0000(3)	0.1394(4)	0.0000(2)	1.0276(1)	-0.0182(8)	0.0000(3)
Prism.Histology	0.0177(7)	0.0698(8)	-0.0760(2)	-0.0325(3)	0.0000(2)	-0.0526(2)	0.0000(3)	0.0040(7)	0.0000(2)	0.0744(6)	0.0289(3)	0.0000(3)
Prism.Stage	0.2156(3)	1.0548(3)	-0.6136(7)	-1.4327(9)	0.5860(1)	-0.4247(6)	0.5422(2)	-0.4777(9)	5.7143(1)	-0.8675(9)	-4.5780(9)	3.9279(1)

**Table 6:** Local variable importance measure for the GINI and MedIncome HPRISM trees. T1, …, T13 indicate terminal node number.

Tree. Variable	T1	T2	T3	T4	T5	T6	T7	T8	T9	T10	T11	T12	T13
MedIncome.Insurance	-0.0006 (7)	-0.0029 (6)	0.0000 (2)	0.0000 (2)	0.0008 (4)	0.0000 (3)							
MedIncome.Surgery	-0.0003 (6)	0.0000 (1)	0.0000 (2)	0.0000 (2)	0.0000 (5)	0.0000 (3)							
MedIncome.Radiation	0.0000 (1)	0.0000 (1)	0.0000 (2)	0.0000 (2)	0.0000 (5)	0.0000(3)							
MedIncome.Chemo	0.0000 (1)	0.0000 (1)	-0.0189 (9)	0.0000 (2)	0.0000 (5)	0.5675 (2)							
MedIncome.Marriage	0.0000 (1)	0.0000 (1)	0.0000 (2)	0.0000 (2)	0.0000 (5)	0.0000 (3)							
MedIncome.Age	-0.0283 (8)	-0.0904 (8)	0.0000 (2)	0.2207 (1)	0.0888 (1)	0.0000 (3)							
MedIncome.Grade	0.0000 (1)	-0.0047 (7)	0.0000 (2)	0.0000 (2)	0.0019 (3)	0.0000 (3)							
MedIncome.Histology	0.000 (1)	0.000 (1)	0.000 (2)	0.000 (2)	0.000 (5)	0.000 (3)							
MedIncome.Stage	-0.0807 (9)	-0.1088 (9)	0.2016 (1)	-0.0014 (9)	0.0039 (2)	2.2372 (1)							
GINI.Insurance	-0.0024 (4)	-0.1309 (5)	0.0750 (3)	0.0000 (2)	0.0095 (5)	0.0000 (3)	0.0575 (6)	0.0000 (3)	0.0788 (4)	0.0211 (5)	0.0389 (3)	0.6991 (5)	0.0000 (3)
GINI.Surgery	0.0298 (8)	-0.0141 (2)	0.0127 (8)	-0.0208 (9)	0.0000 (6)	0.6769 (1)	1.2427 (1)	-0.0150 (9)	0.0174 (8)	0.0000 (7)	0.0133 (6)	0.0824 (8)	-0.0302 (9)
GINI.Radiation	0.0000 (2)	0.0000 (1)	0.0000 (9)	0.0000 (2)	0.0000 (6)	0.0000 (3)	0.0000 (8)	0.0000 (3)	0.0000 (9)	0.0000 (7)	0.0000 (7)	0.0000 (9)	0.0000 (3)
GINI.Chemo	0.0000 (2)	-0.0530 (4)	0.0206 (7)	0.0000 (2)	0.0000 (6)	0.0000 (3)	0.0000 (8)	0.0813 (2)	0.0373 (7)	0.0000 (7)	0.0159 (5)	0.2470 (6)	0.8040 (2)
GINI.Marriage	-0.0069 (7)	-0.2328 (7)	0.1024 (2)	0.0000 (2)	0.0253 (4)	0.0000 (3)	0.0898 (5)	0.0000 (3)	0.1928 (2)	0.0528 (4)	0.0730 (2)	1.0845 (2)	0.0000 (3)
GINI.Age	0.0190 (1)	-0.3765 (8)	0.0538 (4)	0.0000 (2)	0.3224 (1)	0.0000 (3)	0.4426 (2)	0.0000 (3)	0.0810 (3)	0.3458 (1)	-0.1918 (8)	0.9562 (3)	0.0000 (3)
GINI.Grade	-0.0058 (6)	-0.2118 (6)	0.1610 (1)	0.0000 (2)	0.0623 (3)	0.0000 (3)	0.1585 (4)	0.0000 (3)	0.2015 (1)	0.0661 (3)	0.1555 (1)	1.1542 (1)	0.0000 (3)
GINI.Histology	-0.0042 (5)	-0.0418 (3)	0.0349 (6)	0.0000 (2)	-0.0004 (9)	0.0000 (3)	0.0017 (7)	0.0000 (3)	0.0456 (6)	0.0031 (6)	0.0319 (4)	0.2383 (7)	0.0000 (3)
GINI.Stage	-0.0816 (9)	-0.3882 (9)	0.0405 (5)	0.0469 (1)	0.0841 (2)	0.4283 (2)	0.1909 (3)	0.3861 (1)	0.0698 (5)	0.2074 (2)	-0.2709 (9)	0.8305 (4)	3.1025 (1)

Using the GINI HPRISM tree as an example, S4 and S5 Figs (S1 File) show the raw tract level MedIncome values for observations grouped by the HPRISM MedIncome tree terminal nodes (the node numbering system is not important here). Different colors correspond to different terminal nodes and the shading of the color indicates the magnitude of the tract level MedIncome value. All other such raw tract value by tree node plots are available in the S1 File. These plots clearly demonstrate that each terminal node consists of observations from a mixture of tracts and that the number of tracts represented in each terminal node can vary greatly. This feature paves the way for SPADE calculations at each tract based on the methods previously described. S6 Fig (S1 File) shows heat maps of our new SPADEs for tracts across Florida as derived from the PRISM and HPRISM GINI and MedIncome trees. We have plotted tract level z-scores for the estimated weighted ratio in survival times between WNH to BNH for each heat map. With the various HPRISM heat maps, we had to use a robust z-score instead of the usual z-score since the distributions of tract level disparity estimates were heavy-tailed and heavily skewed. This standardization makes the tract SPADE estimates more comparable across heat maps. While these are derived area level measures, they come from models that account for both individual level and area level moderation of racial differences in survival.

**Fig 6:**
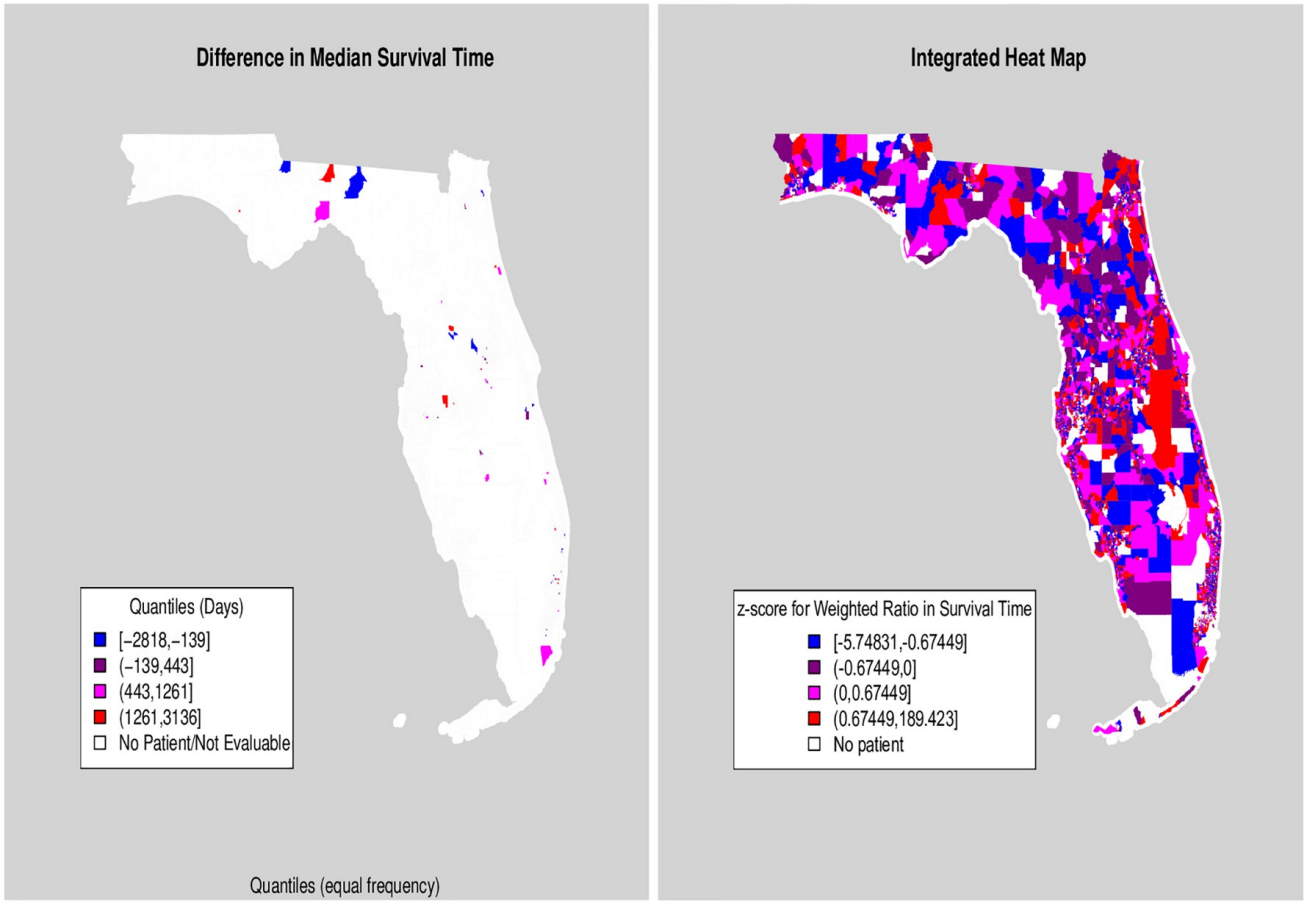
Direct estimate of tract-specific median differences in survival (left). Integrated composite SPADE heat map (right).

There are clear differences between the PRISM and HPRISM plots. The PRISM heat map is heavily concentrated around the middle of the disparity distribution (i.e. purple colored) whereas the HPRISM ones show much more diversity in colors. These are striking indications of hierarchical moderation of race (and by extension, disparity), by tract level variables. Some patterns become apparent. Once again, using MedIncome as a focus, overlaying MedIncome and comparing S4 and S5 Figs in S1 File, regions where MedIncome is high tend to exacerbate WNH:BNH racial disparities with WNH surviving longer on average than BNH as compared to the PRISM SPADE estimates. This effect is most noticeable in large metropolitan regions like Jacksonville, South Florida, and the Tampa/Orlando area. The opposite effect happens for areas with low MedIncome (for example, more rural areas of Florida). Here, the HPRISM SPADE estimates indicate that BNH:WNH survival differences tend to even out as compared to the PRISM SPADE estimates. There also appears to be a clear difference in spatial distribution of SPADEs from the northern part of the state to the south. Similar kinds of pattens can be found by looking at the other HPRISM SPADE heat maps in S7 Fig (S1 File). Fig 6 (right hand plot) puts everything together in a composite heat map showing the combined effect of all social determinants using the approximation described in the Methods section. As a comparison, we show the direct Kaplan-Meier estimate in the left hand plot of Fig 6. For the direct estimate, there are many tracts where the Kaplan-Meier curves do not drop to 50% survival and so a difference between median survivals between racial groups could not be estimated. There are some places where the direct estimate and the SPADE estimate agree and some where disagreements happen. The direct estimates in these tracts tend to have small sample sizes hence these are highly variable and less trustworthy. Importantly, the other thing that is clearly notable when comparing these figures is that there is clear borrowing information happening across tracts in the SPADE estimates which is why so many more tracts have estimates in them. This is actually akin to techniques used in small area estimation [30].

## Discussion

While racial disparities in endometrial cancer survival have previously been studied, the understanding of underlying moderators has been limited by existing statistical methods. Using a new method, this study estimated the moderation of racial disparities in endometrial cancer survival, integrating the hierarchical moderation effects of individual/clinical and social determinants of health in a way that is faithful to the underlying social ecological framework for understanding disparities. Ecological models have been used to study disparities before, but the accompanying quantitative approaches did not allow hierarchical interactions or have not been pursued due to sample size issues [4].

In this study, we specifically made the following important methodological contributions to address these limitations: i) we developed a non-parametric modeling framework for structured moderation (PRISM) which because of the tree-like structure, also yields interpretable models of moderation; ii) this modeling framework allowed for the identification of disparity sub-groups without their apriori specification thereby allowing for the possible lack of homogeneity in disparity in the training dataset; iii) we developed a new measure of local variable importance within the context of the PRISM model; iv) we demonstrated some new uses of data visualization tools to extract interpretable structure from the model and; v) we developed a new estimator (the SPADE) for examining the geographical variation of the model estimated disparities, employing a new type of borrowing strength across census tracts by grouping patients with similar hierarchical moderation effects of race differences as determined by their individual-level determinant variables, allowing more reliable estimates in sparsely populated tracts. In doing so, it’s also capturing variation across grouped tracts, which may likely mimic unmeasured variation within tracts due to using average contextual level information. To the best of our knowledge, no such estimator has been previously developed. We have previously identified a generalization to unit level small area estimation (SAE) [30] using trees [31]. However, much of this work did not consider hierarchical interactions. McConville and Toth developed a tree-based approach for automated selection of post strata when estimating finite population totals but did not deal with small area models [32].

Despite its strengths, there are several important limitations of our study. First, using cancer registry data imposes methodological challenges, including multiple cancer diagnoses, duplicate reports, reporting delays, and misclassification of race [33]. While these can negatively impact model fit and predictive performance, we used a rigorous, unbiased predictive measure and our models retained robust predictive performance. Second, our models do not assess (moderated) race as a causal factor, but as a predictor. There is existing work on causal inference trees, but they have not been generalized to handle the hierarchical moderation required in the social ecological framework [34]. Third, we have based our analysis around a hierarchical moderation framework motivated by sociological ecological theory. While our new models demonstrate good fit to the data, it is possible that other competing models that assume a different structured approximation might also fit the data well. A deeper comparison of different model formulations is forthcoming. Fourth, the binary framework used for surgery, chemotherapy, and radiation variables. Those are primary drivers of survival, based on what type and stage of EC thus making it more complicated to interpret the benefit a more robust modeling approach. Another limitation is the highly selective inclusion criteria. Great care went into eliminating any combination of grade and histology codes that were contradictory (e.g. carcinosarcoma histology and grade 1 nuclear features) and those for which the site of tumor origin was unclear (e.g. adenocarcinoma) or incorrect (e.g. squamous cell carcinoma). While such selectivity likely decreased the sample size and thus potentially the power to identify definitive associations between groups, it was felt that the risk of misclassification bias was too large if a general, unfiltered data set was analyzed. Finally, while the role of genetics is not being modeled here, these determinants may be important moderators given that ecological factors may contribute to epigenetic differences across racial groups.

While the identification and understanding of disparities was our primary goal, our results may inform multilevel interventions that may be used to attenuate disparities and potentially predict effectiveness across neighborhoods [35]. By understanding how area-level factors (often influenced by policy) interact with individual-level factors to determine disparities in endometrial cancer survival, and the fact that these interactions result in pockets of more extreme disparity spatially oriented across the state, one could conceivably design targeted multilevel intervention strategies that could be more effective in reducing disparity.

### Software availability

All code for fitting PRISM and HPRISM models can be downloaded from https://rdrr.io/github/yuhuilin619/prism/.

## Supporting information

S1 Data (GZ)

S1 File (PDF)

## References

[1] Bronfenbrenner U., “Toward an experimental ecology of human development,” *American Psychologist*, vol. 32, p. 513, 1977. doi: 10.1037/0003-066X.32.7.513

[2] Bronfenbrenner U., “*The Ecology of Human Development*, cambridge, ma: Harvard university press,” 1979.

[3] Krieger N., “Theories for social epidemiology in the 21st century: an ecosocial perspective,” *International Journal of Epidemiology*, vol. 20, pp. 668–677, 2001. doi: 10.1093/ije/30.4.668 11511581

[4] Gomez S., Shariff-Marco S., DeRouen M., et al., “The impact of neighborhood social and built environment factors across the cancer continuum: current research, methodological considerations and future directions,” *Cancer*, vol. 121, pp. 2314–2330, 2015. doi: 10.1002/cncr.29345 25847484 PMC4490083

[5] Hiatt R. and Breen N., “The social determinants of cancer: a challenge for transdisciplinary science,” *American Journal of Preventive Medicine*, vol. 35, pp. S141–150, 2008. doi: 10.1016/j.amepre.2008.05.006 18619394 PMC10773976

[6] Lynch S. and Rebbeck T., “Bridging the gap between biologic, individual, and macroenvironmental factors in cancer: a multilevel approach,” *Cancer epidemiology*, *biomarkers & prevention: a publication of the American Association for Cancer Research*, *cosponsored by the American Society of Preventive Oncology*, vol. 22, pp. 485–495, 2013. doi: 10.1158/1055-9965.EPI-13-0010 PMC3645448 23462925

[7] Allard J. and Maxwell G., “Race disparities between black and white women in the incidence, treatment, and prognosis of endometrial cancer,” *Cancer Control*, vol. 16, pp. 53–56, 2009. doi: 10.1177/107327480901600108 19078930

[8] Hill H., Eley J., Harlan L., et al., “Racial differences in endometrial cancer survival: the black/white cancer survival study,” *Obstetrics & Gynecology*, vol. 88, pp. 919–926, 1996. doi: 10.1016/S0029-7844(96)00341-9 8942828

[9] Siegel R., Miller K., and Jemal A., “Cancer statistics,” *CA Cancer J Clin*, vol. 67, pp. 7–30, 2017. doi: 10.3322/caac.21387 28055103

[10] Clarke M., Devesa S., Harvey S., and Wentzensen N., “Hysterectomy-corrected uterine corpus cancer incidence trends and differences in relative survival reveal racial disparities and rising rates of nonendometrioid cancers,” *J Clin Oncol*, vol. 37, pp. 1895–1908, 2019. doi: 10.1200/JCO.19.00151 31116674 PMC6675596

[11] Doll K., Snyder C., and Ford C., “Endometrial cancer disparities: a race-conscious critique of the literature,” *Am J Obstet Gynecol*, vol. 218, pp. 474–482, 2018. doi: 10.1016/j.ajog.2017.09.016 28964822

[12] Madison T., Schottenfeld D., and Baker V., “Cancer of the corpus uteri in white and black women in michigan, 1985?1994: An analysis of trends in incidence and mortality and their relation to histologic subtype and stage,” *Cancer: Interdisciplinary International Journal of the American Cancer Society*, vol. 83, pp. 1546–1554, 1998. doi: 10.1002/(SICI)1097-0142(19981015)83:8<1546::AID-CNCR9>3.0.CO;2-M 9781948

[13] Madison T., Schottenfeld D., James S., et al., “Endometrial cancer: socioeconomic status and racial/ethnic differences in stage at diagnosis, treatment, and survival,” *American Journal of Public Health*, vol. 94, pp. 2104–2111, 2004. doi: 10.2105/ajph.94.12.2104 15569961 PMC1448599

[14] Chia V., Newcomb P., Trentham-Dietz A., et al., “Obesity, diabetes, and other factors in relation to survival after endometrial cancer diagnosis,” *International Journal of Gynecological Cancer*, vol. 17, pp. 441–446, 2007. doi: 10.1111/j.1525-1438.2007.00790.x 17362320

[15] F. Greene, D. Page, I. Fleming, and others eds, “*AJCC Cancer Staging Manual 6th ed*. new york: Springer. american joint committee on cancer,” 2002.

[16] Schlumbrecht M., Baeker-Bispo J., Balise R., et al., “Variation in type ii endometrial cancer risk by hispanic subpopulation: An exploratory analysis,” *Gynecol Oncol*, vol. 147, pp. 329–333, 2017. doi: 10.1016/j.ygyno.2017.09.002 28888539

[17] Hastie T. and Tibshirani R., “Varying coefficient models,” *Journal of the Royal Statistical Society B*, vol. 55, pp. 757–779, 1993.

[18] Stute W., “Consistent estimation under random censorship when covariables are present,” *Journal of Multivariate Analysis*, vol. 45, pp. 89–103, 1993. doi: 10.1006/jmva.1993.1028

[19] Breiman L., Friedman J., Olshen R., and Stone C., “Classification and regression trees, wadsworth & brooks,” 1984.

[20] Novak P., “Checking goodness-of-fit of the accelerated failure time model for survival data,” *WDS’10 Proceedings*, vol. Part I, pp. 189–194, 2010.

[21] Uno H., Cai T., Pencina M., D’Agostino R., and Wei L., “On the c-statistics for evaluating overall adequacy of risk prediction procedures with censored survival data,” *Statistics in Medicine*, vol. 30, pp. 1105–1117, 2011. doi: 10.1002/sim.4154 21484848 PMC3079915

[22] Harrell F., Califf R., Pryor D., Lee K., and Rosati R., “Evaluating the yield of medical tests,” *JAMA*, vol. 247, pp. 2543–2546, 1982. doi: 10.1001/jama.1982.03320430047030 7069920

[23] Ishwaran H., “Variable importance in binary regression trees and forests,” *Electronic Journal of Statistics*, vol. 1, pp. 519–537, 2007. doi: 10.1214/07-EJS039

[24] Andrews D., “Plots of high-dimensional data,” *Biometrics*, vol. 28, pp. 125–136, 1972. doi: 10.2307/2528964

[25] Felsenstein J., “Confidence limits on phylogenies: an approach using the bootstrap,” *Evolution*, vol. 39, pp. 783–791, 1985. doi: 10.1111/j.1558-5646.1985.tb00420.x 28561359

[26] Efron B., Halloran E., and Holmes S., “Bootstrap confidence levels for phylogenetic trees,” *PNAS*, vol. 93, p. 13429, 1996. doi: 10.1073/pnas.93.23.13429 8917608 PMC24110

[27] Zhu L., Sun X., and Bai W., “Nomograms for predicting cancer-specific and overall survival among patients with endometrial cancer: a seer based study,” *Front Oncol*, vol. 10, p. 269, 2020. doi: 10.3389/fonc.2020.00269 32266128 PMC7096479

[28] Miller K., Nogueira L., Mariotto A., et al., “Cancer treatment and survivorship statistics, 2019,” *CA: A Cancer Journal for Clinicians*, vol. 69, pp. 363–385, 2019. 31184787 10.3322/caac.21565

[29] Tejerizo-Garcia A., Jimenez-Lopez J., Munoz-Gonzalez J., et al., “Overall survival and disease-free survival in endometrial cancer: prognostic factors in 276 patients,” *Onco Targets Ther*, vol. 6, pp. 1305–1313, 2013. doi: 10.2147/OTT.S51532 24092993 PMC3787927

[30] Rao J. and Molina I., *Small Area Estimation*, 2nd Ed. Wiley, New York., 2015.

[31] Mendez G. and Lohr S., “Estimating residual variance in random forest regression,” *Computational Statistics & Data Analysis*, vol. 55, pp. 2937–2950, 2011. doi: 10.1016/j.csda.2011.04.022

[32] McConville K. and Toth D., “Automated selection of post-strata using a model-assisted regression tree estimator,” 2017.

[33] Izquierdo J. and Schoenbach V., “The potential and limitations of data from population-based state cancer registries,” *American Journal of Public Health*, vol. 90, pp. 695–698, 2000. doi: 10.2105/ajph.90.5.695 10800415 PMC1446235

[34] Su X., Kang J., Fan J., et al., “Facilitating score and causal inference trees for large observational studies,” *Journ. of Machine Learning Research*, vol. 13, pp. 2955–2994, 2012.

[35] Scholmerich V. and Kawachi I., “Translating the socio-ecological perspective into multilevel interventions: gaps between theory and practice,” *Health Education and Behavior*, vol. 43, pp. 17–20, 2016. doi: 10.1177/1090198115605309 26747715

