# Supplementary material for: Partially Recursively Induced Structured Moderation (PRISM) for modeling racial differences in endometrial cancer survival
Source: PLoS One. 2023 Jan 31;18(1):e0268221. doi: 10.1371/journal.pone.0268221 (PMC9888685; doi:10.1371/journal.pone.0268221)
Supplement: S1 File — (PDF) [file pone.0268221.s002.pdf]

Supplementary Material for "Partially Recursively Induced Structured Moderation (PRISM) for a deeper understanding of the complexity of racial differences in endometrial cancer survival"

J. Sunil Rao,[1,3†*] Erin Kobetz,[2,1,3†] Huilin Yu,[1], Jordan Baeker-Bispo,[1], Zinzi Bailey,[3]

[1]Department of Public Health Sciences, University of Miami
[2] Department of Medicine, University of Miami
[3] Sylvester Comprehensive Cancer Center, University of Miami
[†]Joint first authors

[*]To whom correspondence should be addressed; E-mail: jrao@miami.edu.

October 1, 2019

Additional Tables and Figures for the Results Section

Tables

Table 1: S1: Description of census-tract level social determinants of health examined.

Social Determinant	Definition
MedIncome	Median household income
GINI	GINI coefficient
PercentPov	Percent of individuals living below poverty level
PercentUnEmp	Percent of individuals 16+ in civilian labor force who are unemployed
PercentLowEd	Percent of adults 25+ with less than high school education
PercentCrowd	Percent of housing units with more than 1 resident per room
PercentNoVeh	Percent of housing units with no access to a vehicle
PercentRent	Percent of housing units that are renter-occupied

Table 2: S2: MedIncome HPRISM terminal node parameter estimates. Notice how small the interaction estimates are (columns 4 and 5). This coincides with Figure 5.

Terminal Node Number (from full tree)	Mean Log Survival Time	Intercept	Race Effect	MedIncome (race=WNH)	MedIncome (race=BNH)
2	5.485	6.271	0.663	1.725e-05	-6.173e-08
12	5.028	3.571	21.152	5.381e-05	-6.713e-04
26	5.880	1.355	8.643	1.005e-04	-7.546e-05
108	5.724	8.878	-0.351	-4.415e-05	-1.097e-04
109	5.590	6.415	2.006	5.837e-06	-5.480e-05
55	5.703	6.844	-1.537	4.636e-07	1.419e-05
7	5.875	7.047	-1.508	-4.114e-07	-1.185e-06

Table 3: S3: Local variable importance (Lvimp) table for individual level variables for MedIncome, PercentLowEd and GINI HPRISM models. T1-T7 indicate numberings for the tree terminal nodes. Table column entries are the Lvimp ranks for variables in each terminal node.

Tree.Variable	T1(rank)	T2 (rank)	T3 (rank)	T4 (rank)	T5 (rank)	T6 (rank)	T7 (rank)
MedIncome.Insurance	-0.0040 (3)	-0.0647 (9)	0.4628 (3)	0.1213 (6)	-0.2860 (9)	-0.4858 (9)	1.0081 (2)
MedIncome.Surgery	-0.0087 (6)	0.0148 (3)	0.0563 (8)	0.0689 (7)	-0.0076 (4)	-0.0214 (4)	0.0975 (6)
MedIncome.Radiation	0.0000 (1)	0.0000 (4)	0.0000 (9)	0.0000 (9)	0.0000 (3)	0.0000 (3)	0.0000 (8)
MedIncome.Chemo	0.0000 (1)	-0.0182 (6)	0.1786 (7)	0.0160 (8)	-0.0354 (5)	-0.0584 (5)	-0.0270 (9)
MedIncome.Marriage	-0.0046 (4)	-0.0497 (8)	0.4956 (2)	0.2308 (2)	-0.1366(7)	-0.1740 (7)	0.0084 (7)
MedIncome.Age	-0.1260(9)	0.8984(1)	1.0303(1)	1.1770 (1)	0.6533 (1)	0.4865 (1)	2.2817 (1)
MedIncome.Grade	-0.0096 (7)	-0.0494(7)	0.4239 (4)	0.1896 (4)	-0.1465 (8)	-0.4427 (8)	0.4481 (3)
MedIncome.Histology	-0.0228 (8)	0.1081 (2)	0.2799 (6)	0.2116 (3)	0.0647 (2)	0.0271 (2)	0.3231 (4)
MedIncome.Stage	-0.0080 (5)	-0.0146 (5)	0.3041 (5)	0.1219(5)	-0.0883(6)	-0.1363 (6)	0.2444 (5)
GINI.Insurance	-0.09915)	0.0105 (2)	0.1118(4)				
GINI.Surgery	-0.0235 (2)	0.0000(4)	0.0000(8)				
GINI.Radiation	0.0000 (1)	0.0000 (4)	0.0000 (8)				
GINI.Chemo	-0.0626 (3)	-0.0069 (6)	0.0421 (6)				
GINI.Marriage	-0.1067 (6)	-0.0436 (9)	0.0482 (5)				
GINI.Age	-0.3666 (9)	0.5389 (1)	1.3067 (1)				
GINI.Grade	-0.1204 (7)	0.0002 (3)	0.1678 (3)				
GINI.Histology	-0.0895 (4)	-0.0131 (7)	0.0226 (7)				
GINI.Stage	-0.1300 (8)	-0.0369 (8)	0.1758 (2)				

Figure Captions

Figure 1. S1: FCDS endometrial cancer cohort overall survival by race. The log rank p-value is $p < 0.0001$.

Figure 2. S2: (a) HPRISM PercentCrowd tree; (b) HPRISM PercentNoVeh tree; (c) HPRISM PercentRent tree; (d) HPRISM PercentUnEmp tree

Figure 3. S3: Andrews curves for PRISM and HPRISM models for GINI and MedIncome respectively.

Figure 4. S4: Distribution of raw tract level MedIncome values for observations in each of the terminal nodes for the HPRISM MedIncometree. Different colors correspond to different terminal nodes and the degree of shading corresponds to the magnitude of the tract level MedIncome value.

Figure 5. S5: Distribution of raw tract level MedIncome values for observations in each of the terminal nodes for the HPRISM MedIncome tree. Different colors correspond to different terminal nodes and the degree of shading corresponds to the magnitude of the tract level MedIncome value.

Figure 6. S6: PRISM SPADE heat map (as transformed z-scores) and HPRISM SPADE heat maps for GINI and MedIncome (as transformed robust z-scores). Note how much more *uniformly purple* (i.e from the middle of the distribution) the PRISM estimates are.

Figure 7. S7: HPRISM SPADE heat maps for PercentCrowd, PercentNoVeh, PercentRent and PercentUnEmp (as transformed robust z-scores).

Figures

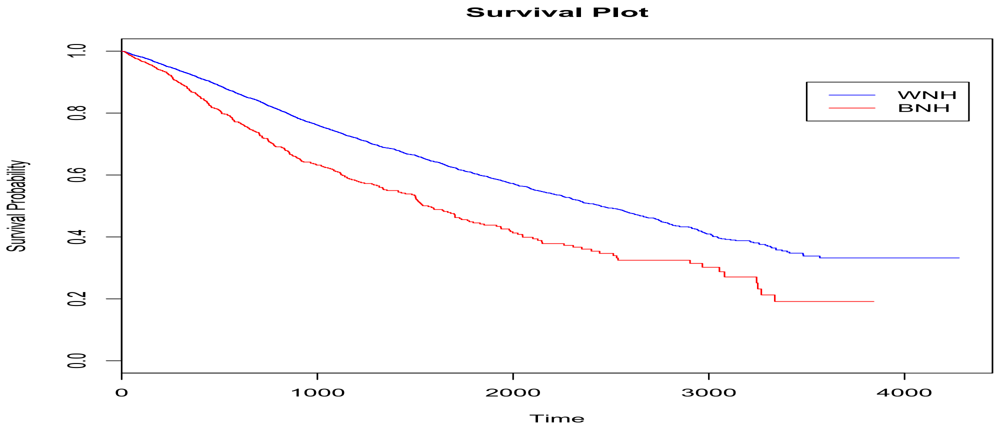

Figure 1: S1: FCDS endometrial cancer cohort overall survival by race. The log rank p-value is $p < 0.0001$.

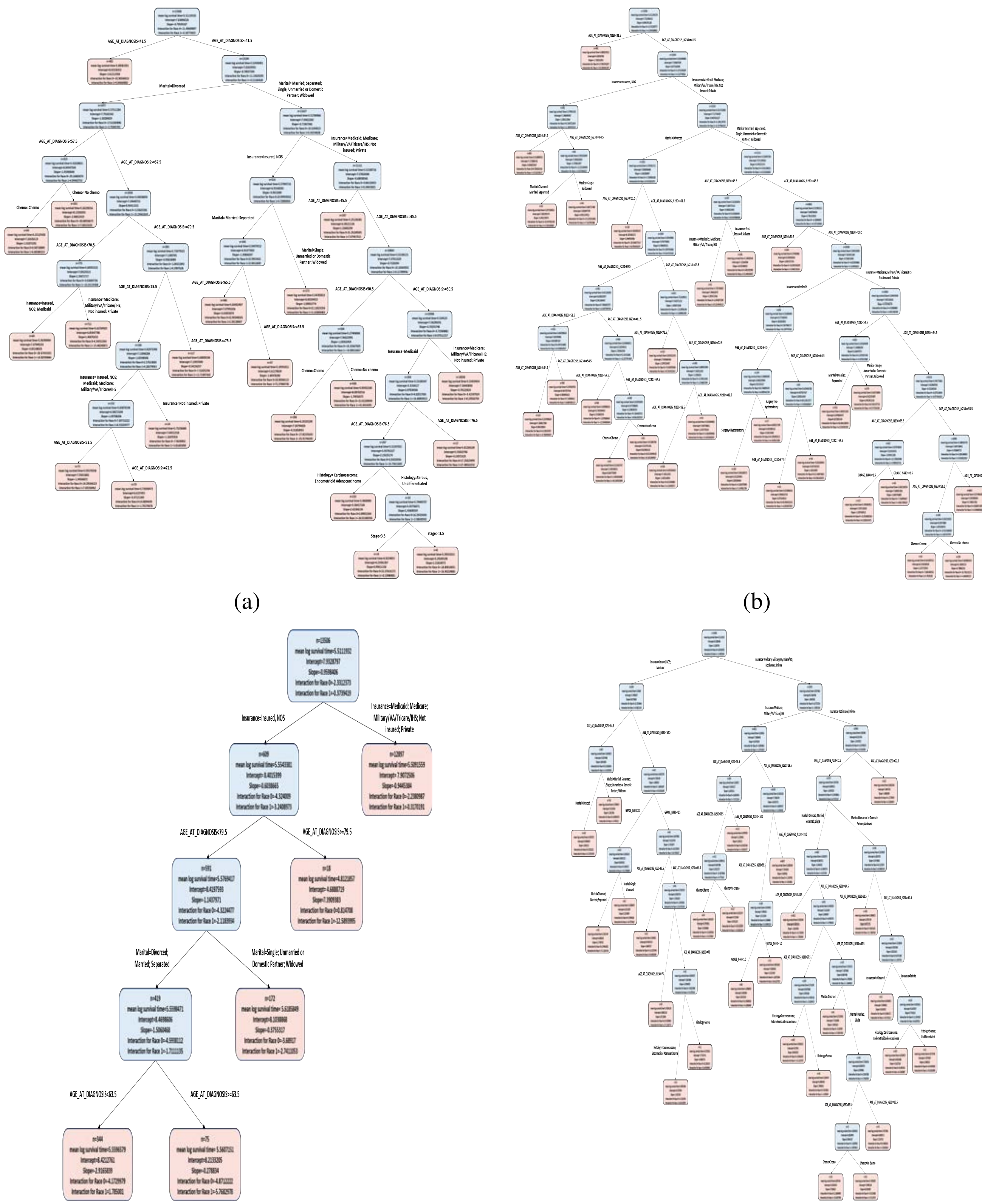

(a) (b) (c) (d)

Figure 2: S2: (a) HPRISM PercentCrowd tree; (b) HPRISM PercentNoVeh tree; (c) HPRISM PercentRent tree; (d) HPRISM PercentUnEmp tree

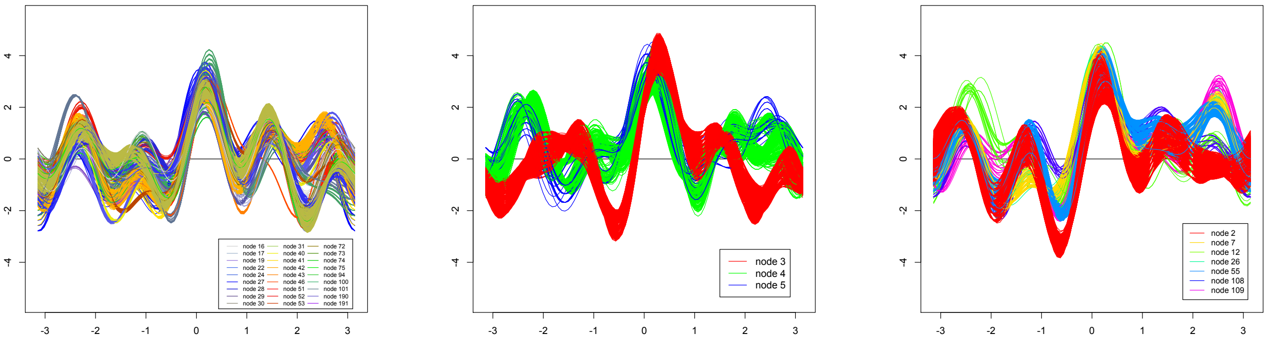

(a) PRISM (b) GINI (c) MedIncome

Figure 3: S3: Andrews curves for PRISM and HPRISM models for GINI and MedIncome respectively.

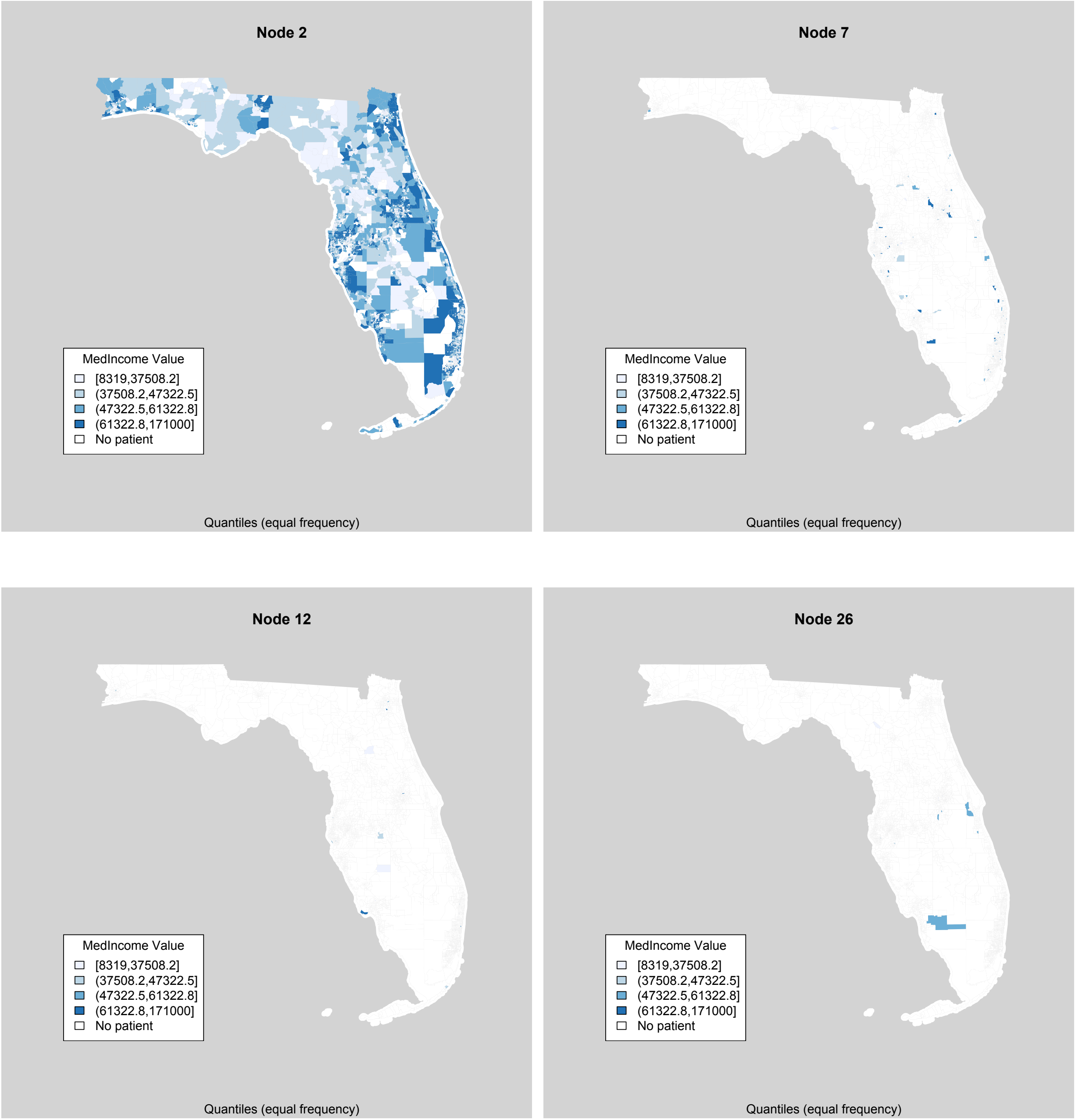

Figure 4: S4: Distribution of raw tract level MedIncome values for observations in each of the terminal nodes for the HPRISM MedIncometree. Different colors correspond to different terminal nodes and the degree of shading corresponds to the magnitude of the tract level MedIncome value.

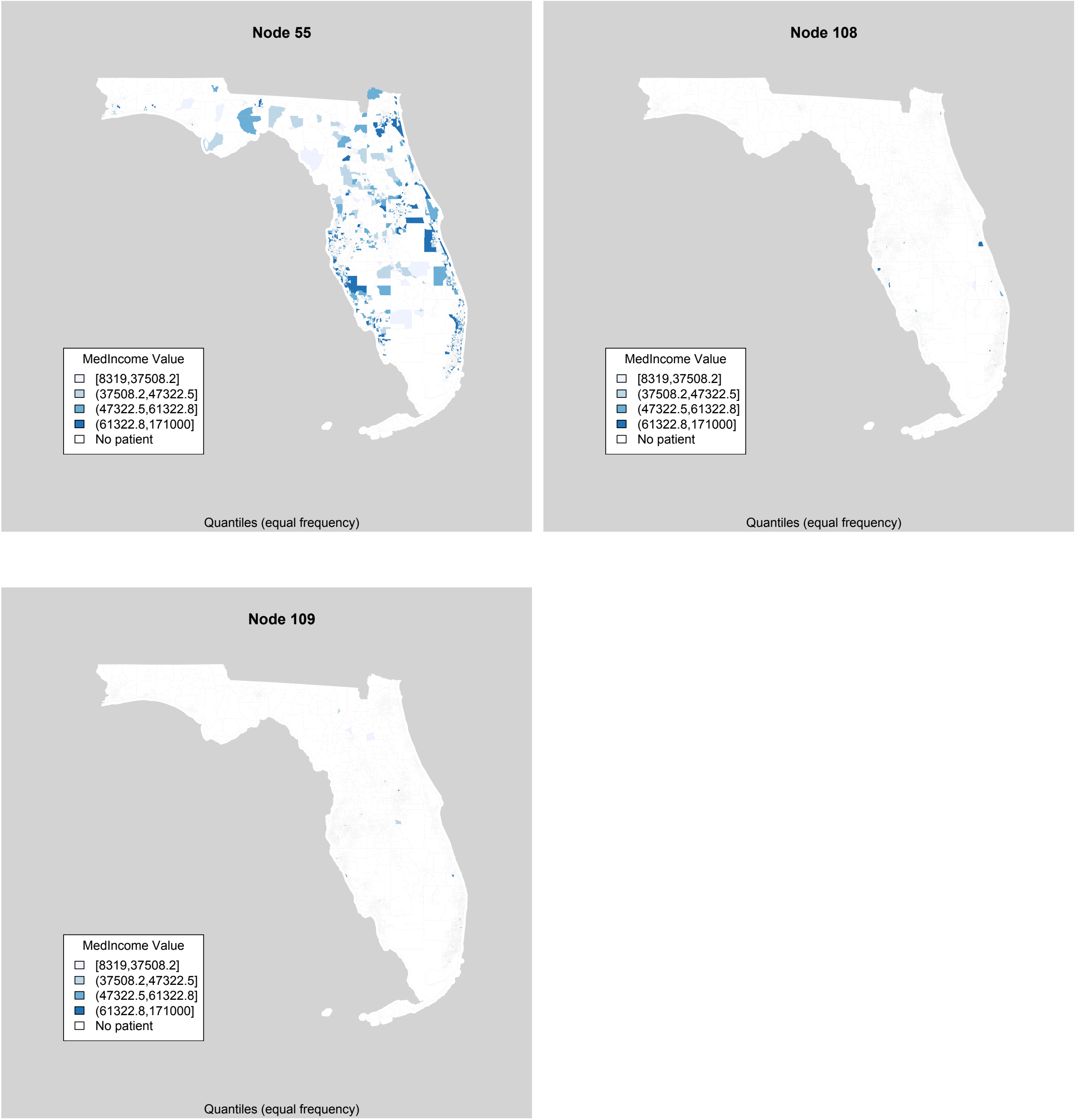

Figure 5: S5: Distribution of raw tract level MedIncome values for observations in each of the terminal nodes for the HPRISM MedIncome tree. Different colors correspond to different terminal nodes and the degree of shading corresponds to the magnitude of the tract level MedIncome value.

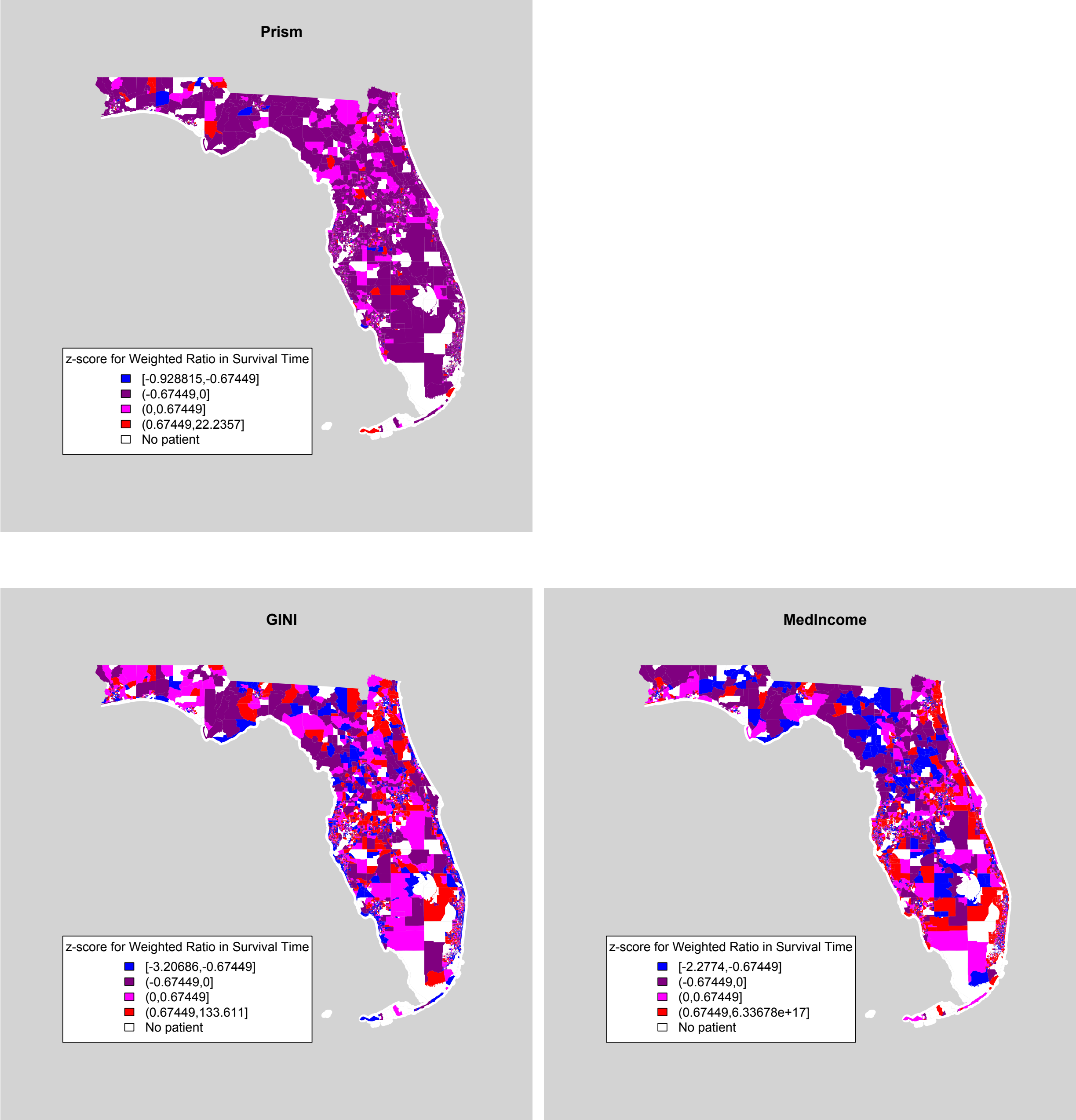

Figure 6: S6: PRISM SPADE heat map (as transformed z-scores) and HPRISM SPADE heat maps for GINI and MedIncome (as transformed robust z-scores). Note how much more *uniformly purple* (i.e from the middle of the distribution) the PRISM estimates are.

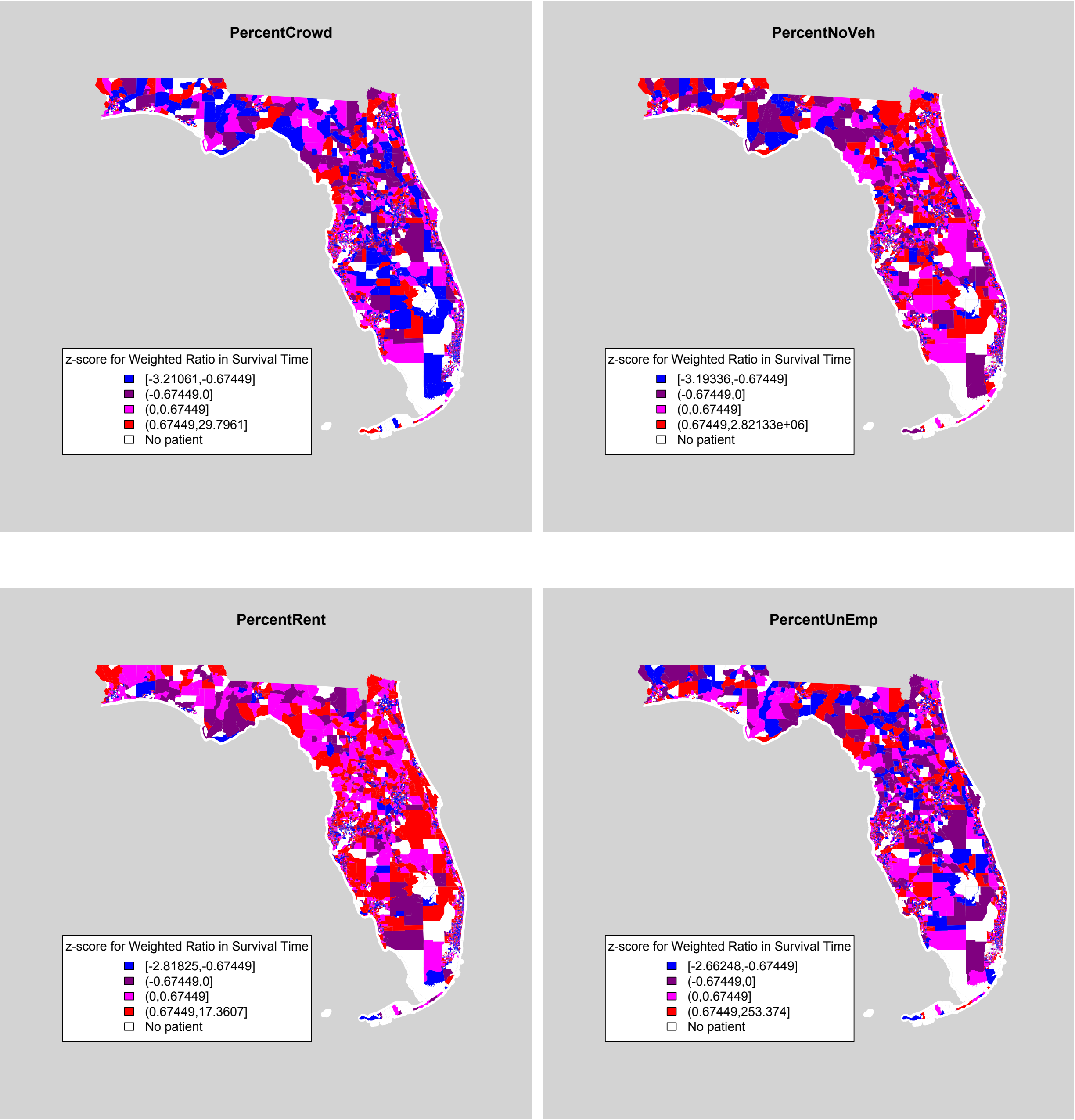

Figure 7: S7: HPRISM SPADE heat maps for PercentCrowd, PercentNoVeh, PercentRent and PercentUnEmp (as transformed robust z-scores).
